# Supplementary material for: Incidence of straight and angulated screw channel following immediate implant placement in esthetic zone: A simulated cone-beam computed tomography study
Source: PLoS One. 2024 Sep 5;19(9):e0308158. doi: 10.1371/journal.pone.0308158 (PMC11376541; doi:10.1371/journal.pone.0308158)
Supplement: Attachment — Submitted filename: Response to Reviewers.docx [file pone.0308158.s002.docx]

To the Editor and reviewers of the PLOS ONE journal

Dear Editor and reviewers,

Thank you for taking the time to review my manuscript. I appreciate your feedback; they were accommodating. I have incorporated all of your suggestions in my revisitation as described in this letter.

Reviewer #1:

1. Manuscript is well written but it can be further improved with adequate proofreading (e.g.paragraph from line 66 to 77). Please do the needful.

Answer: Line 66-70 have been revised in the introduction.

2. Table 2: 'each relation' - can be more specific (e.g.anatomical relation, etc)

Answer: Table 2 and table 3 have been revised and adjusted in page 10 and 11 respectively.

3. Conclusion: The authors state that "the use of angulated screw is essential to increase the probability of screw retained implant prosthesis".

Can the authors explain further on the relevance of this statement in relation to the statement problem and the purpose of this study?

Answer: In our study screw retained implant prosthesis allows for the lowest probability 2.64% (19/720) to perform IIP in esthetic zone. By incorporating angulated screw with 25-degree compensation could increase the probability of screw retained implant prosthesis to 55.42% (399/720). In individual tooth, an angulated screw channel could help increase the probability for screw retained implant prosthesis in central incisor from 7.92% (19/240) to 72.08% (173/240), in lateral incisors from 0% to 50% (120/240), and in canine from 0% to 47.92% (115/240). So, the use of angulated screw channel is essential to increase the probability of screw retained implant prosthesis.

Reviewer #2:

Abstract
1. Line 14: Please specify the number of patients as well.

Answer: 120 patients have been added to the abstract.

2. Line 15: Please specify the method used to come up with the probability of 76.11%. What’s the diameter of the implants? Was angulated screw channel (ASC) included in this calculation?

Answer: 548 teeth from 720 teeth (548/720) can be performed IIP in esthetic zone. The diameter of 3.3, 3.5, 4.1, 4.5 and 5 mm were used in all maxillary anterior teeth (central incisor, lateral incisor, canine). The diameter of 2.9 mm is used in lateral incisor only. The angulated screw channel was included in the calculation of all implant diameter except diameter of 2.9 mm was not included.

3. Line 18: What is “limited restorability angulation for cement retained implant prosthesis (middle half)”? This description is confusing. Please revise.

Answer: It is the most buccal angulation that allow for IIP in esthetic zone in cement retained implant prosthesis, which often in the middle of individual tooth, hence referred to as “middle half”. Even though, there are some possibilities to restore implant in more extreme buccal angulation with implant platform being more apical and palatal, there is an increased risk of complication.

Material and Method
1. Line 90: How did you determine a number of 120 CBCT scans is sufficient for the study?

Answer: Sample size calculation

The purpose of this study is to determine the probability of IIP in esthetic zone using four different implant directions. This design of the study has not been performed yet. So, there will be no similarity in statistical data for this study.

The sample size calculation for estimating proportion will be used as shown in the formula:

d	: margin of error

According to the retrospective study of Ashok Velayudhan et al. in 2020 that the objective related to the current study showed that in a total of 77 implants placed in the anterior maxilla, with 21 of them being immediate implants, 6 (or 7.8%) of 21 immediate implants were placed in the anterior maxilla alone.

The output of the sample size calculation is 114. The 120 patients are sufficient for our study.

2. For the inclusion/exclusion criteria, did you have any criteria to assess if the patient’s anterior teeth were misaligned?

Answer: Upper anterior teeth (canine-canine) misaligned is considered to exclusion criteria, which did not include in the sample size of our study.

3. Line 132: Any citations you could provide to support the use of 4 mm as the threshold?

Answer: The citation has been added to line 132.

4. The authors should elaborate on the methods used to determine the long axis of the tooth, the angulation/direction of the planned implant, etc

Answer: The line drawn from incisal edge to root apex was long axis of the tooth.

The point between incisal edge to CEJ was marked as point M. The point between the center of the most prominent point of cingulum to the incisal edge was marked as point S.

- In traditional cement retained implant prosthesis (incisal edge), long axis of implant will coincide with incisal edge.

- In traditional screw retained implant prosthesis (screw retained), long axis of implant will coincide with point S.

- In limit angulation for angulated screw channel (angle screw), long axis of implant will coincide at the point that an angle up to 25° from traditional screw retained.

- In limit restorability angulation for cement retained implant prosthesis (middle half), long axis of implant will coincide with point M.

Results

1. How did you decide the implant length and diameter for central incisors, lateral incisors and canines? For example, is it practical to place a 5mm-diameter implant at a canine location, or to place a 16mm- or even 18mm-long implant?

Answer: In our study, the guideline for implant positioning includes, positioning implant platform at 3 mm apical and 2 mm palatal to gingival margin, a minimum distant of 1.5 mm from adjacent teeth, implant engaging at least 4 mm of apical bone, with at least 2 mm of buccal bone to implant gap to accommodate the grafting material to minimize bone remodeling. In our study was digital implant planning to determine the probability of IIP in esthetic zone by using different diameter (2.9, 3.3, 3.5, 3.75, 4.0, 4.5, 5 mm) and length (6, 8, 10, 12, 14, 16, 18 mm). To determine the diameter and length, select the smallest diameter and length and gradually increase the diameter and length until reaching a size where the implant exist in alveolar housing while respecting aforementioned guideline. It is possible to placed implant with diameter 5 mm and length 16-18 mm in canine since it is allowed by the guideline. However, it does not explicitly state that using these diameter and length is the first choice for IIP in esthetic zone.

Discussion
1. There are two very similar studies published in 2022 and 2023. The authors must provide comments on these two studies, and elaborate on how the current study can add more information to the currently available evidence.
Erin K Edmondson, Pedro M Trejo, Nikolaos Soldatos, Robin L Weltman. The ability to screw-retain single implant-supported restorations in the anterior maxilla: A CBCT analysis. J Prosthet Dent. 2022 Sep;128(3):443-449.
Joseph Y K Kan, Kitichai Rungcharassaeng, Pongrapee Kamolroongwarakul, Guo-Hao Lin, Hiroyuki Matsuda, Shi Yin, Hom-Lay Wang, Dennis Tarnow, Jaime L Lozada. Frequency of screw-retained angulated screw channel single crown following immediate implant placement and provisionalization in the esthetic zone: A cone beam computed tomography study/ Clin Implant Dent Relat Res. 2023 Oct;25(5):789-794.

Answer: In Erin K Edmondson study was virtual implant planning by Simplant® Pro17.01; Dentsply Sirona in screw retained implant direction using straight or an angled screw channel abutment with different angulation (5°, 10°, 15°). The process is not IIP, therefore the method to placed implant was different from our study. The implant diameter for each site was fix based on standards for each tooth type (canine and central incisor 4.3 mm, lateral incisor 3.5 mm). The implant length was 10 mm for all implant.

In Joseph Y K Kan study investigate the probability of using straight screw-channel screw-retained restoration for immediate implant placement in maxillary anterior teeth. The method of this study was different from our study. The mid sagittal image of each maxillary teeth with vertical and horizontal scale bars was screen-captured and transferred to Keynote. The digital template for 3.5 mm diameter was used in central incisor and lateral incisor. The digital template for 4.3 mm diameter was used in central incisor and canine. The implant length was 13, 15 and 18 mm. Additional line added to digital template as a guide for IIP. All CBCT images were classified as contraindicated or indicated by two examiners.

In our study was virtual implant planning by Implant Studio® to determine the probability of IIP in esthetic zone. The directions of implant that added for more than the two mentioned study (Erin K Edmondson and Joseph Y K Kan) were cement retained implant prosthesis, angulated screw channel at the 25° angulation and the middle of individual tooth. Various size of diameter (2.9, 3.3, 3.5, 3.75, 4.0, 4.5, 5 mm) and length (6, 8, 10, 12, 14, 16, 18 mm) were included in our study to promote adaptability and suitability in IIP in esthetic zone.

2. The authors should comment on the limitations of the current study.

Answer: In our study, to standardized the method, implant planning is limited to static predetermined angulation to anatomical relation and implant size. This could limited the probability as clinician can increase the depth of implant placement, modified implant angulation within restorable position. Future study is suggested to study the effect of probability associated with dynamic implant planning.

Finally, I appreciate your feedback. They are accommodating and explicitly more reliable to my work. I am open to revising and improving my manuscript based on your suggestions. Thank you again for your time and consideration.

I look forward to your reply,

Sincerely,

Pranai Nakaparksin, DDS, MSD, FACP, FRCD(c)

Department of Advanced General Dentistry, Faculty of Dentistry, Mahidol University.

6 Yothi Street, Thung Phaya Thai, Rachathewi, Bangkok 10400 Thailand

Tel. +662 200 7853

Mobile. +66818088111

E-mail: Pranai.nak@mahidol.ac.th
