# Supplementary material for: Incidence of straight and angulated screw channel following immediate implant placement in esthetic zone: A simulated cone-beam computed tomography study
Source: PLoS One. 2024 Sep 5;19(9):e0308158. doi: 10.1371/journal.pone.0308158 (PMC11376541; doi:10.1371/journal.pone.0308158)
Supplement: Attachment — Submitted filename: Response to Reviewers.docx [file pone.0308158.s003.docx]

To the Editor and reviewers of the PLOS ONE journal

Dear Editor and reviewers,

Thank you for taking the time to review my manuscript. I appreciate your feedback; they were accommodating. I have incorporated all of your suggestions in my revisitation as described in this letter.

**To Editor:**

- All references have been reviewed and corrected to ensure completeness.

- Citation [37.	Erin K Edmondson, Pedro M Trejo, Nikolaos Soldatos, Robin L Weltman. The ability to screw-retain single implant-supported restorations in the anterior maxilla: A CBCT analysis. J Prosthet Dent. 2022 Sep;128(3):443-449. Available from: <https://doi.org/10.1016/j.prosdent.2021.01.004>] has been added to line 273-274 in the discussion part of manuscript.

- Citation [38.	Joseph Y K Kan, Kitichai Rungcharassaeng, Pongrapee Kamolroongwarakul, Guo-Hao Lin, Hiroyuki Matsuda, Shi Yin, Hom-Lay Wang, Dennis Tarnow, Jaime L Lozada. Frequency of screw-retained angulated screw channel single crown following immediate implant placement and provisionalization in the esthetic zone: A cone beam computed tomography study/ Clin Implant Dent Relat Res. 2023 Oct;25(5):789-794. Available from: <https://doi.org/10.1111/cid.13227>] has been added to line 274 and 278 in the discussion part of manuscript.

**Reviewer #2:**

**1. Answer for using angulated screw channel of not: 548 teeth from 720 teeth (548/720) can be performed IIP in esthetic zone. The diameter of 3.3, 3.5, 4.1, 4.5 and 5 mm were used in all maxillary anterior teeth (central incisor, lateral incisor, canine). The diameter of 2.9 mm is used in lateral incisor only. The angulated screw channel was included in the calculation of all implant diameter except diameter of 2.9 mm was not included.**

- The angulated screw channel was included in the calculation of all implant diameter except diameter of 2.9 mm was not included. The following sentence has been added to line 121-122 in the material and method part of manuscript. The remaining sentences are already included in line 118-121 in the material and method part of manuscript.

**2. Answer for clarifying “limited restorability angulation for cement retained implant prosthesis": It is the most buccal angulation that allow for IIP in esthetic zone in cement retained implant prosthesis, which often in the middle of individual tooth, hence referred to as “middle half”. Even though, there are some possibilities to restore implant in more extreme buccal angulation with implant platform being more apical and palatal, there is an increased risk of complication**

- The “middle half” has been explained for better comprehension and understanding in line 254-256 in the discussion part of manuscript.

**3. Answer for sample size calculation:
The purpose of this study is to determine the probability of IIP in esthetic zone using four different implant directions. This design of the study has not been performed yet. So, there will be no similarity in statistical data for this study.
The sample size calculation for estimating proportion will be used as shown in the formula (in Response to reviewers):
According to the retrospective study of Ashok Velayudhan et al. in 2020 that the objective related to the current study showed that in a total of 77 implants placed in the anterior maxilla, with 21 of them being immediate implants, 6 (or 7.8%) of 21 immediate implants were placed in the anterior maxilla alone.
The output of the sample size calculation is 114. The 120 patients are sufficient for our study. --> This must be mentioned in the manuscript.**

- This retrospective study of Ashok Velayudhan et al. has been mentioned and cited in line 92-93 in the material and method part of manuscript.

**4. Answer for misaligned teeth as an exclusion criterion: Upper anterior teeth (canine-canine) misaligned is considered to exclusion criteria, which did not include in the sample size of our study.**

- Misaligned upper anterior teeth has been added to exclusion criteria in line 104 in the material and method part of manuscript.

**5.Answer to elaborate on the methods used to determine the long axis of the tooth, the angulation/direction of the planned implant:
The line drawn from incisal edge to root apex was long axis of the tooth.
The point between incisal edge to CEJ was marked as point M. The point between the center of the most prominent point of cingulum to the incisal edge was marked as point S.
- In traditional cement retained implant prosthesis (incisal edge), long axis of implant will coincide with incisal edge.
- In traditional screw retained implant prosthesis (screw retained), long axis of implant will coincide with point S.
- In limit angulation for angulated screw channel (angle screw), long axis of implant will coincide at the point that an angle up to 25° from traditional screw retained.
- In limit restorability angulation for cement retained implant prosthesis (middle half), long axis of implant will coincide with point M.**

- All following sentences have been added to line 126-135 in the material and method part of manuscript.

**6. Discussion**

- The two mentioned studies have been added and compared to our study to line 273-289 in the discussion part of manuscript.

**7. Answer related to the study limitation: In our study, to standardized the method, implant planning is limited to static predetermined angulation to anatomical relation and implant size. This could limited the probability as clinician can increase the depth of implant placement, modified implant angulation within restorable position. Future study is suggested to study the effect of probability associated with dynamic implant planning.**

- All following sentences have been added to line 290-294 in the discussion part of manuscript.

Finally, I appreciate your feedback. They are accommodating and explicitly more reliable to my work. I am open to revising and improving my manuscript based on your suggestions. Thank you again for your time and consideration.

I look forward to your reply,

Sincerely,

Pranai Nakaparksin, DDS, MSD, FACP, FRCD(c)

Department of Advanced General Dentistry, Faculty of Dentistry, Mahidol University.

6 Yothi Street, Thung Phaya Thai, Rachathewi, Bangkok 10400 Thailand

Tel. +662 200 7853

Mobile. +66818088111

E-mail: Pranai.nak@mahidol.ac.th
