# Supplementary material for: Incidence of straight and angulated screw channel following immediate implant placement in esthetic zone: A simulated cone-beam computed tomography study
Source: PLoS One. 2024 Sep 5;19(9):e0308158. doi: 10.1371/journal.pone.0308158 (PMC11376541; doi:10.1371/journal.pone.0308158)
Supplement: Attachment — Submitted filename: Response to Reviewers final .docx [file pone.0308158.s005.docx]

To the Editor and reviewers of the PLOS ONE journal

Dear Editor and reviewers,

Thank you for taking the time to review my manuscript. I appreciate your feedback; they were accommodating. I have incorporated all of your suggestions in my revisitation as described in this letter.

**Reviewer #3:**

**Abstract:**

**“Statement of problem: What are the incidences of immediate implant placement (IIP) in the esthetic zone, using four different implant directions?” - It is wrongly developed.
- The methodology was weakly presented**

**Introduction:**

**- This session is long.**

Answer: All suggestions have been applied and incorporated into the abstract section of manuscript.

**Material and Method:**

**- there is a time problem in the study “The sample criteria for this study were patients who had CBCT scans of the anterior maxilla at the Oral and Maxillofacial Radiology clinic, Faculty of Dentistry, Mahidol University performed during January 2012 – August 2022. CBCT images were performed by 3D Accuitomo 170 (J. 91 Morita®, Japan) and randomly selected for evaluation on 9 June 2021.”**

Answer: The sample criteria for this study were patients who had CBCT scans of the anterior maxilla at the Oral and Maxillofacial Radiology clinic, Faculty of Dentistry, Mahidol University performed during January 2012 – August 2020. CBCT images were performed by 3D Accuitomo 170 (J. 91 Morita®, Japan) and randomly selected for evaluation on 9 June 2021.

**- Exclusion criteria is incomplete**

Answer: - “CBCT images that were distorted or blurred” have been added in exclusion criteria.

**- Statistics was poorly presented**

Answer: This study is descriptive study without comparison group. Descriptive statistics were used to present the probability of IIP in the esthetic zone as percentages.

**Results
- What was the foundation/parameter to support the probability suggested? “The distribution of SRP with probability of IIP are shown in Table 1."**

Answer: The mid-sagittal images were used to classify SRP with respect to the alveolar housing according to Kan et al. [Kan JY, Roe P, Rungcharassaeng K, Patel RD, Waki T, Lozada JL, et al. Classification of sagittal root position in relation to the anterior maxillary osseous housing for immediate implant placement: a cone beam computed tomography study. Int J Oral Maxillofac Implants. 2011 Jul-Aug;26(4):873-6.]

In table 1 show distribution of SRP with probability of IIP as percentages.

In this study have 120 CBCT scans, which focus on upper anterior teeth (720 teeth).

SRP class l have 633 teeth, which perform IIP 533 teeth.

The distribution of SRP class l is 633/720*100 = 87.91%. The probability of IIP in SRP class l is 533/633*100 = 84.20%.

SRP class ll have 20 teeth, which perform IIP 14 teeth.

The distribution of SRP class ll is 20/720*100 = 2.78%. The probability of IIP in SRP class ll is 14/20*100 = 70%.

SRP class lll have 3 teeth, which perform IIP 1 teeth.

The distribution of SRP class lll is 3/720*100 = 0.42%. The probability of IIP in SRP class lll is 1/3*100 = 33.33%.

SRP class lV have 64 teeth, which perform IIP 0 teeth.

The distribution of SRP class lV is 64/720*100 = 8.89%. The probability of IIP in SRP class lV is 0/64*100 = 0%.

**Conclusion
- I suggest to rethink and adjust it after revision.**

Answer: The conclusion section has been revised.

**Reviewer #4:**

**1. Title needs to be improved**

Answer: The title has been improved.
**2. Abstract: The problem statement is not clear and is almost the same as what is mentioned in the purpose.**

Answer: The problem statement has been improved: the demand for immediate implant placement (IIP) in the esthetic zone is rapidly increasing. Despite the treatment’s benefits, the sagittal root position often dictates implant angulation, commonly necessitating the use of cement-retained restorations. This study investigates the impact of angulated screw channel on IIP in the esthetic zone.
**3. Introduction: A. In my opinion, you can shorten your introduction by removing unnecessary details; B. Your introduction needs to be strengthened. You need to add a paragraph of other studies that used simulations like yours and describe their findings in a very short and meaningful way. C. Some references were missing; D. Add your study hypothesis.**

Answer:

A. The content in the introduction section has been revised to more concise, following the recommendations.

D. This study is descriptive study without comparison group, so there is no study hypothesis.
**4. Material: A. Please specify the population?, which ethnicity? Age (Mean age)? Gender? B. Figure 1 showed misaligned anterior central incisor. One of your exclusion criteria is misaligned teeth, so how come you have included this in your study. This is contradicting and may be translated into bias in your study. Please comment on this. C. In your figure 3, I noticed that there are some issues in your alignment of your Implant. It is clearly evident to the readers in this figure that you are not following exactly Point M or Point S when placing your implants. Even the incisal edge is not followed 100%.
The points mentioned in figure 2 are not exactly followed in figure 3**
**This can affect all your study results and can be considered invalid outcomes. Please comment on this.**

Answer:

A. Details regarding the population have been added to the material and method section.

B. The old image presented in Fig 1 is a CBCT scan section as described in the caption of Fig 1 (In the axial view, the implant was positioned at the center of the selected tooth. In the panoramic view, the implant was aligned parallel to the long axis of tooth. In the coronal view, the implant was rotated parallel to the long axis of tooth. The mid-sagittal image, which was been sectioned at the bucco-lingual aspect of the tooth, was used to measure and plan of IIP). However, it was not used in the planning of IIP and was not included in this study. The image has been revised and replaced for Fig 1.

C. The placement of dental implant at each site will be conducted as described in the material and method section. The position of point M, point S and point P will vary depending on each individual tooth. Nevertheless, there may be some placements that deviate from the reference points in Fig 2. However, this does not affect the outcome of this study, as the determination of whether a tooth is suitable for IIP is based on the implant being surrounded by bone, assessed on an area basis rather than specific points. The image has been revised and replaced for Fig 3.

Finally, I appreciate your feedback. They are accommodating and explicitly more reliable to my work. I am open to revising and improving my manuscript based on your suggestions. Thank you again for your time and consideration.

I look forward to your reply,

Sincerely,

Pranai Nakaparksin, DDS, MSD, FACP, FRCD(c)

Department of Advanced General Dentistry, Faculty of Dentistry, Mahidol University.

6 Yothi Street, Thung Phaya Thai, Rachathewi, Bangkok 10400 Thailand

Tel. +662 200 7853

Mobile. +66818088111

E-mail: Pranai.nak@mahidol.ac.th
