# Supplementary material for: Incidence of straight and angulated screw channel following immediate implant placement in esthetic zone: A simulated cone-beam computed tomography study
Source: PLoS One. 2024 Sep 5;19(9):e0308158. doi: 10.1371/journal.pone.0308158 (PMC11376541; doi:10.1371/journal.pone.0308158)
Supplement: Attachment — Submitted filename: PONE-D-23-29819-reviewer comments.docx [file pone.0308158.s004.docx]

PLOS ONE

# Incidence of straight and angulated screw channel following immediate implant placement in esthetic zone: A CBCT study

--Manuscript Draft--

| Manuscript Number: | PONE-D-23-29819R2 |
| --- | --- |
| Article Type: | Research Article |
| Full Title: | Incidence of straight and angulated screw channel following immediate implant placement in esthetic zone: A CBCT study |
| Short Title: | Incidence of straight and angulated screw channel following immediate implant placement. |
| Corresponding Author: | Pranai Nakaparksin, DDS, MSD Mahidol University Faculty of Dentistry Bangkok, Rachathewi THAILAND |
| Keywords: | cone beam computed tomography; Dental implant; immediate implant placement; esthetic zone; sagittal root position; angle screw; cement retained implant prosthesis; screw retained implant prosthesis; digital planning; buccal bone thickness; implant length; implant diameter |
| Abstract: | Abstract  Statement of problem: What are the incidences of immediate implant placement (IIP) in the esthetic zone, using four different implant directions?  Purpose: The purpose of this cone-beam computed tomography (CBCT) study was to determine the probability of IIP in the esthetic zone, using four different implant directions.  Materials and methods: A retrospective review of CBCT images was conducted as a part of this study and accessed for research purpose on 9 June 2021. The midsagittal images of maxillary anterior teeth (right canine to left canine) were input into an implant planning software (Implant Studio®). Bone Level X Straumann® implant (BLX 3.5, 3.75, 4.0, 4.5, 5.0 mm) and Bone Level Tapered implant (BLT 2.9, 3.3 mm) were selected for this study. The diameter of 2.9 mm was used in lateral incisor only. The frequency distribution and probability of IIP were recorded and compared among all maxillary anterior teeth.  Results: CBCT images from 720 teeth (120 patient) were evaluated. We found the overall probability of IIP in the esthetic zone is 76.11% (548/720). Four different implant directions were evaluated in this study, with the following results; traditional screw retained implant prosthesis (screw retained), traditional cement retained implant prosthesis (incisal edge), limited angulation for angulated screw channel (angle  screw) and limited restorability angulation for cement retained implant prosthesis (middle half) are 2.64% (19/720), 26.11% (188/720), 55.42% (399/720) and 38.75%  (279/720) respectively.  Conclusion: Angulated screw channels is imperative in increasing the probability of IIP for screw retained implant prosthesis.  Clinical implications: For communication with the patient to determine treatment options for replacement of failing teeth in esthetic zone. To understand how that angulated screw channel can increase the chance for IIP in the dental esthetic zone. |
| Order of Authors: | Watcharapon Radomsutthisarn, DDS |
|  | Pranai Nakaparksin, DDS, MSD |
|  | Kornkamol Kretapirom, DDS, PhD |
| Response to Reviewers: | To Editor:   - All references have been reviewed and corrected to ensure completeness. - Citation [37.Erin K Edmondson, Pedro M Trejo, Nikolaos Soldatos, Robin L Weltman. The ability to screw-retain single implant-supported restorations in the anterior maxilla: A CBCT analysis. J Prosthet Dent. 2022 Sep;128(3):443-449. Available from: https://doi.org/10.1016/j.prosdent.2021.01.004] has been added to line 273-274 in the discussion part of manuscript. - Citation [38.Joseph Y K Kan, Kitichai Rungcharassaeng, Pongrapee |

*Powered by Editorial Manager® and ProduXion Manager® from Aries Systems Corporation*

|  | Kamolroongwarakul, Guo-Hao Lin, Hiroyuki Matsuda, Shi Yin, Hom-Lay Wang, Dennis Tarnow, Jaime L Lozada. Frequency of screw-retained angulated screw channel single crown following immediate implant placement and provisionalization in the esthetic zone: A cone beam computed tomography study/ Clin Implant Dent Relat Res. 2023 Oct;25(5):789-794. Available from: https://doi.org/10.1111/cid.13227] has been added to line 274 and 278 in the discussion part of manuscript.  Reviewer #2:  1. Answer for using angulated screw channel of not: 548 teeth from 720 teeth (548/720) can be performed IIP in esthetic zone. The diameter of 3.3, 3.5, 4.1, 4.5 and 5 mm were used in all maxillary anterior teeth (central incisor, lateral incisor, canine). The diameter of 2.9 mm is used in lateral incisor only. The angulated screw channel was included in the calculation of all implant diameter except diameter of 2.9 mm was not included.  - The angulated screw channel was included in the calculation of all implant diameter except diameter of 2.9 mm was not included. The following sentence has been added to line 121-122 in the material and method part of manuscript. The remaining sentences are already included in line 118-121 in the material and method part of manuscript.  2. Answer for clarifying “limited restorability angulation for cement retained implant prosthesis": It is the most buccal angulation that allow for IIP in esthetic zone in cement retained implant prosthesis, which often in the middle of individual tooth, hence referred to as “middle half”. Even though, there are some possibilities to restore implant in more extreme buccal angulation with implant platform being more apical and palatal, there is an increased risk of complication  - The “middle half” has been explained for better comprehension and understanding in line 254-256 in the discussion part of manuscript.  3. Answer for sample size calculation:  The purpose of this study is to determine the probability of IIP in esthetic zone using four different implant directions. This design of the study has not been performed yet. So, there will be no similarity in statistical data for this study.  The sample size calculation for estimating proportion will be used as shown in the formula (in Response to reviewers):  According to the retrospective study of Ashok Velayudhan et al. in 2020 that the objective related to the current study showed that in a total of 77 implants placed in the anterior maxilla, with 21 of them being immediate implants, 6 (or 7.8%) of 21 immediate implants were placed in the anterior maxilla alone.  The output of the sample size calculation is 114. The 120 patients are sufficient for our study. --> This must be mentioned in the manuscript.  - This retrospective study of Ashok Velayudhan et al. has been mentioned and cited in line 92-93 in the material and method part of manuscript.  4. Answer for misaligned teeth as an exclusion criterion: Upper anterior teeth (canine- canine) misaligned is considered to exclusion criteria, which did not include in the sample size of our study.  - Misaligned upper anterior teeth has been added to exclusion criteria in line 104 in the material and method part of manuscript.   5. Answer to elaborate on the methods used to determine the long axis of the tooth, the angulation/direction of the planned implant:   The line drawn from incisal edge to root apex was long axis of the tooth.  The point between incisal edge to CEJ was marked as point M. The point between the center of the most prominent point of cingulum to the incisal edge was marked as point S.   - In traditional cement retained implant prosthesis (incisal edge), long axis of implant will coincide with incisal edge. - In traditional screw retained implant prosthesis (screw retained), long axis of implant will coincide with point S. - In limit angulation for angulated screw channel (angle screw), long axis of implant will coincide at the point that an angle up to 25° from traditional screw retained. - In limit restorability angulation for cement retained implant prosthesis (middle half), long axis of implant will coincide with point M. - All following sentences have been added to line 126-135 in the material and method part of manuscript.   6. Discussion  - The two mentioned studies have been added and compared to our study to line 273- 289 in the discussion part of manuscript. |
| --- | --- |

*Powered by Editorial Manager® and ProduXion Manager® from Aries Systems Corporation*

|  | 7. Answer related to the study limitation: In our study, to standardized the method, implant planning is limited to static predetermined angulation to anatomical relation and implant size. This could limited the probability as clinician can increase the depth of implant placement, modified implant angulation within restorable position. Future study is suggested to study the effect of probability associated with dynamic implant planning.  - All following sentences have been added to line 290-294 in the discussion part of manuscript.  Finally, I appreciate your feedback. They are accommodating and explicitly more reliable to my work. I am open to revising and improving my manuscript based on your suggestions. Thank you again for your time and consideration.  I look forward to your reply, |
| --- | --- |
| Additional Information: |  |
| Question | Response |
| Financial Disclosure  Enter a financial disclosure statement that describes the sources of funding for the work included in this submission. Review the [submission guidelines](http://journals.plos.org/plosone/s/submission-guidelines#loc-financial-disclosure-statement) for detailed requirements. View published research articles from [*PLOS ONE*](http://journals.plos.org/plosone/) for specific examples.  This statement is required for submission and will appear in the published article if the submission is accepted. Please make sure it is accurate. | The authors received no specific funding for this work. |
| Unfunded studies  Enter: *The author(s) received no specific funding for this work.*  Funded studies  Enter a statement with the following details:   - Initials of the authors who received each award - Grant numbers awarded to each author - The full name of each funder - URL of each funder website - Did the sponsors or funders play any role in the study design, data collection and analysis, decision to publish, or preparation of the manuscript? - NO - Include this sentence at the end of   your statement: *The funders had no role in study design, data collection and analysis, decision to publish, or preparation of the manuscript.*   - YES - Specify the role(s) played. |  |
| * typeset |  |

*Powered by Editorial Manager® and ProduXion Manager® from Aries Systems Corporation*

| Competing Interests  Use the instructions below to enter a competing interest statement for this submission. On behalf of all authors, disclose any [competing interests](http://journals.plos.org/plosone/s/competing-interests) that could be perceived to bias this work—acknowledging all financial support and any other relevant financial or non- financial competing interests.  This statement is required for submission and will appear in the published article if the submission is accepted. Please make sure it is accurate and that any funding sources listed in your Funding Information later in the submission form are also declared in your Financial Disclosure statement.  View published research articles from  [*PLOS ONE*](http://journals.plos.org/plosone/) for specific examples. | All authors including, Dr. Pranai Nakaparksin, Dr. Watcharapon Radomsutthisarn, Dr. Kornkamol Kretapirom have declared that no competing interests exist. |
| --- | --- |
| NO authors have competing interests  Enter: *The authors have declared that no competing interests exist.*  Authors with competing interests  Enter competing interest details beginning with this statement:  *I have read the journal's policy and the authors of this manuscript have the following competing interests: [insert competing interests here]* |  |
| * typeset |  |
| Ethics Statement  Enter an ethics statement for this submission. This statement is required if the study involved:   - Human participants - Human specimens or tissue - Vertebrate animals or cephalopods | - Faculty of Dentistry/Faculty of Pharmacy, Mahidol University, Institutional Review Board is in full compliance with International Guidelines for Human Research Protection such as Declaration of Helsinki, the Belmont Report, CIOMS Guidelines and the International Conference on Harmonization in Good Clinical Practice (lCH-GCP)   - COA.No.MU-DT,/PY-IRB 2021/052.0906   - Our research does not require the implementation of a consent form since it is a retrospective study on cone beam computed tomography (CBCT) images for digital implant planing. However, there will be a data anonymization process to ensure patient information is securely protected and used exclusively for the purposes of this research |

*Powered by Editorial Manager® and ProduXion Manager® from Aries Systems Corporation*

| - Vertebrate embryos or tissues - Field research   Write "N/A" if the submission does not require an ethics statement.  General guidance is provided below. Consult the [submission guidelines](http://journals.plos.org/plosone/s/submission-guidelines#loc-guidelines-for-specific-study-types) for detailed instructions. Make sure that all information entered here is included in the Methods section of the manuscript. | project. |
| --- | --- |

*Powered by Editorial Manager® and ProduXion Manager® from Aries Systems Corporation*

| Format for specific study types  Human Subject Research (involving human participants and/or tissue)   - Give the name of the institutional review board or ethics committee that approved the study - Include the approval number and/or a statement indicating approval of this research - Indicate the form of consent obtained (written/oral) or the reason that consent was not obtained (e.g. the data were analyzed anonymously)   Animal Research (involving vertebrate animals, embryos or tissues)   - Provide the name of the Institutional Animal Care and Use Committee (IACUC) or other relevant ethics board that reviewed the study protocol, and indicate whether they approved this research or granted a formal waiver of ethical approval - Include an approval number if one was obtained - If the study involved *non-human primates*, add *additional details* about animal welfare and steps taken to ameliorate suffering - If anesthesia, euthanasia, or any kind of animal sacrifice is part of the study, include briefly which substances and/or methods were applied   Field Research  Include the following details if this study involves the collection of plant, animal, or other materials from a natural setting:   - Field permit number - Name of the institution or relevant body that granted permission |  |
| --- | --- |
|  |  |
| Data Availability  Authors are required to make all data underlying the findings described fully available, without restriction, and from the time of publication. PLOS allows rare exceptions to address legal and ethical concerns. See the [PLOS Data Policy](http://journals.plos.org/plosone/s/data-availability) and [FAQ](http://journals.plos.org/plosone/s/data-availability#loc-faqs-for-data-policy) for detailed information. | Yes - all data are fully available without restriction |

*Powered by Editorial Manager® and ProduXion Manager® from Aries Systems Corporation*

| A Data Availability Statement describing where the data can be found is required at submission. Your answers to this question constitute the Data Availability Statement and will be published in the article, if accepted. |  |
| --- | --- |
| Important: Stating ‘data available on request from the author’ is not sufficient. If your data are only available upon request, select ‘No’ for the first question and explain your exceptional situation in the text box. |  |
| Do the authors confirm that all data underlying the findings described in their manuscript are fully available without restriction? |  |
| Describe where the data may be found in full sentences. If you are copying our sample text, replace any instances of XXX with the appropriate details.   - If the data are held or will be held in a public repository, include URLs, accession numbers or DOIs. If this information will only be available after acceptance, indicate this by ticking the box below. For example: *All XXX files are available from the XXX database (accession number(s) XXX, XXX.*). - If the data are all contained within the manuscript and/or Supporting Information files, enter the following: *All relevant data are within the manuscript and its Supporting Information files.* - If neither of these applies but you are able to provide details of access elsewhere, with or without limitations, please do so. For example:   *Data cannot be shared publicly because of [XXX]. Data are available from the XXX Institutional Data Access / Ethics Committee (contact via XXX) for researchers who meet the criteria for access to confidential data.*  *The data underlying the results presented in the study are available from (include the name of the third party* | All relevant data are within the manuscript and its Supporting Information files. |

*Powered by Editorial Manager® and ProduXion Manager® from Aries Systems Corporation*

| *and contact information or URL).*   - This text is appropriate if the data are owned by a third party and authors do not have permission to share the data.   * typeset |  |
| --- | --- |
| Additional data availability information: | Tick here if the URLs/accession numbers/DOIs will be available only after acceptance of the manuscript for publication so that we can ensure their inclusion before publication. |

*Powered by Editorial Manager® and ProduXion Manager® from Aries Systems Corporation*

<https://www.editorialmanager.com/pone/download.aspx?id=35851017&guid=3b76f55b-bc14-4ebd-a8df-ba585944f2a3&scheme=1>Manuscript	[Click here to access/download;Manuscript;Manuscript.docx](https://www.editorialmanager.com/pone/download.aspx?id=35851017&guid=3b76f55b-bc14-4ebd-a8df-ba585944f2a3&scheme=1)

1

**Incidence of straight and angulated screw channel following immediate implant placement in esthetic zone: a CBCT study**

Watcharapon Radomsutthisarn, DDS ^1¶^, Pranai Nakaparksin, DDS, MSD, FACP, FRCD(c)

^1&*,^ ^#a^, Kornkamol Kretapirom DDS, PhD ^2&^

1 Department of Advanced General Dentistry, Faculty of Dentistry, Mahidol University, Bangkok, Thailand,
2 Department of Oral and Maxillofacial Radiology, Faculty of Dentistry, Mahidol University,

Bangkok, Thailand

#a Current address: Department of Advanced General Dentistry, Faculty of Dentistry, Mahidol University, Bangkok, Thailand

**Corresponding Authors:**

Email: [Pranai.nak@mahidol.ac.th](mailto:Pranai.nak@mahidol.ac.th)

¶These authors contributed equally to this work. &These authors also contributed equally to this work.

1

2 **Abstract**
3 **Statement of problem:** What are the incidences of immediate implant placement (IIP) in the
4 esthetic zone, using four different implant directions?
5 **Purpose:** The purpose of this cone-beam computed tomography (CBCT) study was to
6 determine the probability of IIP in the esthetic zone, using four different implant directions.
7 **Materials and methods:** A retrospective review of CBCT images was conducted as a part of
8 this study and accessed for research purpose on 9 June 2021. The midsagittal images of
9 maxillary anterior teeth (right canine to left canine) were input into an implant planning
10 software (Implant Studio®). Bone Level X Straumann® implant (BLX 3.5, 3.75, 4.0, 4.5, 5.0
11 mm) and Bone Level Tapered implant (BLT 2.9, 3.3 mm) were selected for this study. The
12 diameter of 2.9 mm was used in lateral incisor only. The frequency distribution and
13 probability of IIP were recorded and compared among all maxillary anterior teeth.
14 **Results:** CBCT images from 720 teeth (120 patient) were evaluated. We found the overall
15 probability of IIP in the esthetic zone is 76.11% (548/720). Four different implant directions
16 were evaluated in this study, with the following results; traditional screw retained implant
17 prosthesis (screw retained), traditional cement retained implant prosthesis (incisal edge),
18 limited angulation for angulated screw channel (angle screw) and limited restorability
19 angulation for cement retained implant prosthesis (middle half) are 2.64% (19/720), 26.11%
20 (188/720), 55.42% (399/720) and 38.75% (279/720) respectively.
21 **Conclusion:** Angulated screw channels is imperative in increasing the probability of IIP for
22 screw retained implant prosthesis.
23 **Clinical implications:** For communication with the patient to determine treatment options
24 for replacement of failing teeth in esthetic zone. To understand how that angulated screw
25 channel can increase the chance for IIP in the dental esthetic zone.
26 **Introduction**
27 Immediate implant placement (IIP) following tooth extraction has become a common
28 clinical procedure for the replacement of failing teeth. However, placement of dental implants
29 in the maxillary anterior esthetic zone is challenging for clinicians, due to the patients’ esthetic
30 expectations and several risk factors that affect overall outcome of the treatment. Therefore,
31 for desired esthetic results and tooth function when performing an IIP [1, 2], this procedure
32 requires adequate information (clinical examination, radiographic examination, cone beam
33 computed tomography (CBCT)).
34 Previous studies indicated that the morphological bone changes immediately following
35 tooth extraction. As a result, the alveolar bone and subsequent implant placement can be
36 affected [3-6]. These human studies measured the width of the buccal bone walls of the
37 extraction sockets in the esthetic zone and determined that the mean width of the buccal bony
38 wall in maxillary anterior teeth was 1mm. The majority of the buccal bony walls (87.2%) had
39 a width ≤ 1 mm, while only 2.6% of the walls measured 2 mm wide [7]. In addition, in the

the changes in the vertical bone at the time of implant

study of Spray et al. [8] measured

40

41 insertion and compared these changes to the thickness of the facial bones. The findings of this
42 study revealed that when the facial bone reaches a thickness of 1.8 mm to 2 mm, bone loss
43 significantly decreased. The results showed that the optimal “critical thickness” value for
44 minimizing implant issues and facial bone loss in the facial plate, was approximately 2 mm [8].
45 In these studies, the buccal bone wall in maxillary anterior teeth was 1 mm, and most of them
46 had a width of ≤ 1 mm. Therefore, the recommended prerequisite for bone graft material is a
47 minimum of 2 mm distance between the implant and the internal part of the buccal bone wall.
48 This should be enough to limit midfacial mucosal recession in the future [9].
49 It is important to accurately determine buccal bone thickness, palatal bone thickness,
50 and faciopalatal root diameter to select the appropriate implant diameter and length, in order to
51 prevent unfavorable esthetic consequences following morphological bone changes in the facial
52 aspect. For example, when placing a larger diameter implant, the vascular supply to the buccal
53 plate is compromised, which increases the risk of resorption and recession particularly in the
54 midfacial region. However, when using a smaller diameter implant this can result in the tooth
55 having lower primary stability [10, 11]. When considering which implant length to use, if the
56 implant length is too long, it will invade critical structures such as the inferior alveolar nerve
57 or maxillary sinus, thus generating more trauma. If implant length is too short, it will reduce
58 osteointegration into the alveolar bone, thus reducing in primary stability [12]. Because IIP
59 requires bone engagement at apical area 4-5 mm beyond the tooth socket apex [9], implant
60 length should be considered preoperatively.
61 A preoperative CBCT scan provides valuable information about the site when placing
62 implants in the esthetic zone, including the current buccal and palatal plate width, expected
63 implant diameter and length, and sagittal root position (SRP) [13-15]. Therefore, a preoperative
64 CBCT scan is highly recommended and provides much needed advanced radiographs for
65 proper treatment planning of IIP in the esthetic zone.
66 Restoration on implant can be either screw retained restoration or cement retained
67 restoration [16]. However, cement retained restorations have more advantage in esthetics since
68 they can provide more suitable emergence angle [17] without showing screw access hole. So,
69 the cement retained restorations are better options for concealing the access hole and are practical
70 to use in esthetic zone. Nevertheless, the main drawback of the cement retained restoration is
71 difficulty in cleaning excess cement when performing subgingival margin [18]. While, in
72 screw retained was good in retrievability and not presented excess cement [19]. However,
73 screw access hole will be compromised esthetic. Where the screw access is visible, screw
74 retained can’t be an option. Cement retained restorations are favorable because they allow for
75 more implant positions [16]. Angulated screw channel allows the screw access hole to be
76 placed with an angle up to 25° from long axis of implant. Thus, using an angulated screw
77 channel to avoid the screw access hole, allows fabricating the screw retained restoration
78 without compromising esthetic results [20].
79 The purpose of this study was to determine the probability of IIP in the esthetic zone,
80 using four different implant directions, traditional screw retained implant prosthesis (screw
81 retained), traditional cement retained implant prosthesis (incisal edge), limited angulation for
82 angulated screw channel (angle screw), and limited restorability angulation for cement retained
83 implant prosthesis (middle half).
84 **Material and method**
85 **Patient selection**
86 This retrospective study was approved by the Institutional Review Board (IRB) of the
87 Faculty of Dentistry/Faculty of Pharmacy, Mahidol University (IRB 2021/052.0906). The
88 sample criteria for this study were patients who had CBCT scans of the anterior maxilla at the
89 Oral and Maxillofacial Radiology clinic, Faculty of Dentistry, Mahidol University performed
90 during January 2012 – August 2022. CBCT images were performed by 3D Accuitomo 170 (J.
91 Morita®, Japan) and randomly selected for evaluation on 9 June 2021. The sample size
92 calculation for estimating proportion will be used with retrospective study of Asok Velayudhan
93 et al [21], which the objective related to our study. The sample size calculation is 120 CBCT
94 scans
95 Inclusion criteria were as follows:
96 - CBCT must have field of view (FOV) 6 cm. x 6 cm. (voxel size 0.125 mm) or FOV 8 cm. x
97 8 cm. (voxel size 0.160 mm.).
98 - The patient must be at least 18 years old at the time of CBCT scan.
99 - The patient must have all maxillary anterior teeth (canine to canine).
100 Exclusion criteria were as follows:
101 **-** Radiographic evidence of periapical inflammatory lesion, periodontitis, severe root
102 resorption, trauma, or bone pathology.
103 - Radiographic evidence of artifacts and orthodontic appliances.
104 - Upper anterior teeth were misaligned.
105 **Data reconstruction from CBCT images and digital simulation**
106 CBCT volumetric data was exported to DICOM files and reconstructed by using
107 Implant Studio® (3Shape®, Denmark). For the axial view, the implant was positioned at the
108 center of the selected tooth. In the panoramic view, the implant was aligned parallel to the
109 long axis of the tooth. In the coronal view, the implant was rotated to be parallel to the long
110 axis of the tooth. The mid-sagittal image, which is located at the bucco-lingual side of the
111 tooth, was used to measure all parameters (Fig 1).

**Fig 1 User interface of Implant Studio® software showing the implant placement at**

112

113

**area 13**.

114 The mid-sagittal of the selected tooth was used for the measurement of the buccal bone
115 thickness, palatal bone thickness, and faciopalatal root dimension at the bone crest level.

JY

SRP

116

was classified with respect to the alveolar housing according to Kan

et al. [13].

117 **Implant planning and determining of implant diameter and length**
118 Implant was planned by Implant Studio® (3Shape®, Denmark) using Bone level X
119 Straumann® implant (BLX 3.5, 3.75, 4.0, 4.5, 5.0 mm) (Straumann®, Switzerland) and Bone
120 Level Tapered implant (BLT 2.9, 3.3 mm) (Straumann®, Switzerland). BLT 2.9 mm was used
121 in lateral incisor only. The angulated screw channel was included in the calculation of all
122 implant diameter except diameter of 2.9 mm was not included.
123 To achieve restorative driven, an implant should be placed 3 mm apical and 2 mm
124 palatal to gingival margin [22]. Thus, the 3 mm line apical to gingival margin and a 2 mm
125 line, which perpendicular to the first line were created. The end point was marked as a
126 position of the most buccal aspect of implant platform point P as shown in figure 2. The line drawn from incisal
127 edge to root apex was long axis of the tooth. The point between incisal edge to CEJ was
128 marked as point M. The point between the center of the most prominent point of cingulum to
129 the incisal edge was marked as point S. The implant component points and lines are shown in Fig
131 2.

132 **Fig 2 The component points of the implant are marked in mid-sagittal images.**
133 The implant was positioned in four different directions (Fig 3) as follows: screw
134 retained, incisal edge, angle screw and middle half. In traditional cement retained implant prosthesis
136 (incisal edge), long axis of implant will coincide with incisal edge. In traditional screw
138 retained implant prosthesis (screw retained), long axis of implant will coincide with point S.
140 In limited angulation for angulated screw channel (angle screw), long axis of implant will
142 coincide at the point that an angle up to 25° from traditional screw retained. In limited
144 restorability angulation for cement retained implant prosthesis (middle half), long axis of
147 implant will coincide with point M.
149 **Fig 3 The four different implant directions in this study.**
150 To determine the implant diameter and length, the smallest implant diameter and length
151 were selected. The implant length was increased until engaging a minimum of 4 mm in distance
152 from the apical to root apex [9], which is considered as the first implant position and increased
153 until the apex of implant is positioned 1 mm away from critical structures. After that, increased
154 the next size implant diameter until it reached the maximum, which calculated by faciopalatal
155 root dimension minus two and repeated protocol for the implant length.
156 All measurements (the buccal bone thickness, palatal bone thickness, and faciopalatal
157 root dimension) for the implant planning process were performed by one examiner. The
158 intraexaminer reliability was assessed by repeating the measurement on 10 randomly selected
159 CBCT images 1 month after the first measurement by intra-class correlation coefficient (ICC).
160 Descriptive statistics were used to report the probability of IIP in the esthetic zone
161 using four different implant directions as percentages.
162 **Results**
163 120 CBCT images from maxillary anterior teeth (240 central incisors, 240 lateral
164 incisors, 240 canines) in 120 patients (39 males and 81 females) with a mean age of 35 years
165 old (range: 21 to 77 years old) were evaluated in this study. The ICC of this study is 0.885
166 (good reliability).
167 The result revealed that 84.58 % (609/720) of anterior maxillary teeth had buccal bone
168 of less than 1 mm. The SRP with probability of IIP are as follow: class I 87.91% (633/720)
169 with 84.20% (533/633), class II 2.78% (20/720) with 70% (14/20), class III 0.42% (3/720) with
170 33.33% (1/3) and class IV 8.89% (64/720) with 0% respectively.
171 The distribution of SRP with probability of IIP are shown in Table 1.
172 The probability of IIP in all maxillary anterior teeth are 76.11% (548/720), with consist
173 of central incisor 82.50% (198/240), lateral incisor 64.58% (155/240) and canine 81.25%
174 (195/240). In this study, an individual tooth can have multiple implant positions. The
175 probability of IIP for each relation (10 relations) is shown in Table 2.
176 The probability of IIP in screw retained, incisal edge, angle screw and middle half are

168	2.64% (19/720), 26.11% (188/720), 55.42% (399/720) and 38.75% (279/720) respectively.

169 The probability of IIP in each direction is shown in Table 3.
170 Central incisor can position implant with diameter of 3.3 mm, 3.5 mm, 3.75 mm, 4.0

171	mm and 4.5 mm at 81.67% (196/240), 68.33% (164/240), 54.58% (131/240), 40.83% (98/240)

172 and 5% (12/240) respectively and length of 10 mm, 12 mm, 14 mm, 16 mm and 18 mm at
173 22.5% (54/240), 62.92% (151/240), 80% (192/240), 73.33% (176/240) and 54.58% (131/240)
174 respectively.
175 Lateral incisor can position implant with diameter of 2.9 mm, 3.3 mm, 3.5 mm, 3.75

176	mm and 4.0 mm at 57.92% (139/240), 51.25% (123/240), 36.25% (87/240), 26.67% (64/240)

177 and 15% (36/240) respectively and length of 10 mm, 12 mm, 14 mm, 16 mm and 18 mm at
178 6.67% (16/240), 30% (72/240), 56.67% (136/240), 43.33% (104/240) and 30.83% (74/240)
179 respectively.

| 180 | Canine can position implant with diameter of 3.3 mm, 3.5 mm, 3.75 mm, 4.0 mm, 4.5 |
| --- | --- |
| 181 | mm and 5.0 mm at 73.33% (176/240), 77.92% (187/240), 75.42% (181/240), 71.25% |
| 182 | (171/240), 55% (132/240) and 23.75% (57/240) respectively and length of 10 mm, 12 mm, 14 |
| 183 | mm, 16 mm and 18 mm at 7.5% (18/240), 25.42% (61/240), 49.58% (119/240), 58.75% |
| 184 | (141/240) and 55% (132/240) respectively. |
| 185 | The distribution and the percentages of the diameter and length, which were used for |
| 186 | IIP are shown in Table 4. |
| 187 |  |

| **Table 1 Distribution of sagittal root position** (**SRP**) **with probability of IIP in esthetic zone** | | | | | | | | | | | | |
| --- | --- | --- | --- | --- | --- | --- | --- | --- | --- | --- | --- | --- |
| **SRP** | **Class I** | | | **Class II** | | | **Class III** | | | **Class IV** | | |
| **Distribution** | **633**/**720** (**87.91%**) | | | **20**/**720** (**2.78%**) | | | **3**/**720** (**0.42%**) | | | **64**/**720** (**8.89%**) | | |
| **Probability** | **533**/**633** (**84.20%**) | | | **14**/**20** (**70%**) | | | **1**/**3** (**33.33%**) | | | **0**/**64** (**0%**) | | |
|  | **Central** | **Lateral** | **Canine** | **Central** | **Lateral** | **Canine** | **Central** | **Lateral** | **Canine** | **Central** | **Lateral** | **Canine** |
| **Distribution** | **213**(**88.75%**) | **183**(**76.25%**) | **237**(**98.75%**) | **9**(**3.75%**) | **11**(**4.58%**) | **-** | **1**(**0.42%**) | **2**(**0.83%**) | **-** | **17**(**7.08%**) | **44**(**18.34%**) | **3**(**1.25%**) |

188

189

190

| **Table 2 Detailed probability of IIP by angulation groups and tooth type** | | | | |
| --- | --- | --- | --- | --- |
|  | **Central incisor** (**240 teeth**) | **Lateral incisor** (**240 teeth**) | **Canine** (**240 teeth**) | **Total** (**720 teeth**) |
| **1. Incisal edge** (**I**) | **24**(**10%**) | **29**(**12.08%**) | **7**(**2.92%**) | **60**(**8.33%**) |
| **2. Screw retained** (**S**) | **1**(**0.42%**) | **-** | **-** | **1**(**0.14%**) |
| **3. Angle screw** (**A**) | **57**(**23.75%**) | **52**(**21.66%**) | **21**(**8.75%**) | **130**(**18.06%**) |
| **4. Middle half** (**M**) | **1**(**0.42%**) | **6**(**2.5%**) | **73**(**30.41%**) | **80**(**11.11%**) |
| **5. I, S** | **8**(**3.33%**) | **-** | **-** | **8**(**1.11%**) |
| **6. I, A** | **47**(**19.58%**) | **10**(**4.17%**) | **6**(**2.5%**) | **63**(**8.75%**) |
| **7. A, M** | **26**(**10.83%**) | **45**(**18.75%**) | **78**(**32.5%**) | **149**(**20.69%**) |
| **8. I, S, A** | **7**(**2.92%**) | **-** | **-** | **7**(**0.97%**) |
| **9. I, A, M** | **24**(**10%**) | **13**(**5.42%**) | **10**(**4.17%**) | **47**(**6.53%**) |
| **10. I, S, A, M** | **3**(**1.25%**) | **-** | **-** | **3**(**0.42%**) |
| **Total** | **198/240**(**82.5%**) | **155**/**240**(**64.58%**) | **195**(**81.25%**) |  |

191

192

193

194

195

| **Table 3 Summary of IIP probability by angulation groups and tooth type** | | | | |
| --- | --- | --- | --- | --- |
| **Angulation** | **Central incisor** (**240 teeth**) | **Lateral incisor** (**240 teeth**) | **Canine** (**240 teeth**) | **Total** (**720 teeth**) |
| **Incisal edge**  **1, 5, 6, 8, 9, 10** | **113**(**47.08%**) | **52**(**21.67%**) | **23**(**9.58%**) | **188**(**26.11%**) |
| **Screw retained**  **2, 5, 8, 10** | **19**(**7.92%**) | **0** | **0** | **19**(**2.64%**) |
| **Angle screw**  **3, 6, 7, 8, 9, 10** | **164**(**68.33%**) | **120**(**50%**) | **115**(**47.92%**) | **399**(**55.42%**) |
| **Middle half**  **4, 7, 9, 10** | **54**(**22.5%**) | **64**(**26.67%**) | **161**(**67.08%**) | **279**(**38.75%**) |

196

197

198 1. I = incisal edge, 2. S = screw, 3. A = angle, 4. M = middle, 5. I, S = incisal edge and screw, 6. I, A = incisal edge and angle, 7. A, M = angle
199 and middle, 8. I, S, A = incisal edge, screw and angle, 9. I, A, M = incisal edge, angle and middle, 10. I, S, A, M = incisal edge, screw, angle and
200 middle

| **Table 4 Distributions of diameter and length of implant used in immediate implant placement in esthetic zone** | | | | | | | | | | | | | |
| --- | --- | --- | --- | --- | --- | --- | --- | --- | --- | --- | --- | --- | --- |
| **Diameter** | | | | | | | **Length** | | | | | | |
|  | **Central**  (**240 teeth**) | | **Lateral**  (**240 teeth**) | | **Canine**  (**240 teeth**) | |  | **Central**  (**240 teeth**) | | **Lateral**  (**240 teeth**) | | **Canine**  (**240 teeth**) | |
| **2.9 mm** | **-** | **-** | **139** | **57.92%** | **-** | **-** | **6 mm** | **-** | **-** | **-** | **-** | **-** | **-** |
| **3.3 mm** | **196** | **81.67%** | **123** | **51.25%** | **176** | **73.33%** | **8 mm** | **-** | **-** | **-** | **-** | **-** | **-** |
| **3.5 mm** | **164** | **68.33%** | **87** | **36.25%** | **187** | **77.92%** | **10 mm** | **54** | **22.5%** | **16** | **6.67%** | **18** | **7.5%** |
| **3.75 mm** | **131** | **54.58%** | **64** | **26.67%** | **181** | **75.42%** | **12 mm** | **151** | **62.92%** | **72** | **30%** | **61** | **25.42%** |
| **4.0 mm** | **98** | **40.83%** | **36** | **15%** | **171** | **71.25%** | **14 mm** | **192** | **80%** | **136** | **56.67%** | **119** | **49.58%** |
| **4.5 mm** | **12** | **5%** | **-** | - | **132** | **55%** | **16 mm** | **176** | **73.33%** | **104** | **43.33%** | **141** | **58.75%** |
| **5 mm** | **-** | **-** | **-** | **-** | **57** | **23.75%** | **18 mm** | **131** | **54.58%** | **74** | **30.83%** | **132** | **55%** |

201

202

203

204

205

206 **Discussion**
207 The success criteria for immediate implant placement (IIP) in the esthetic zone
208 including non-acute infection areas, presence of thick gingival biotype, engaging sufficient
209 apical bone, with intact buccal bone [9]. Recent studies have shown that on average, the buccal
210 bone in anterior maxillary teeth was less than 1 mm [7, 23]. This study found similar results
211 with the majority of anterior maxillary teeth, 84.58% (609/720), having less than 1 mm of
212 buccal bone thickness. Canine teeth 29.17% (70/240), were found most frequently to have a
213 buccal bone thickness greater than 1 mm, followed by the lateral incisors and central incisors
214 11.25% (27/240) and 5.83% (14/240) respectively.
215 There was a 12.5% (90/720) probability of IIP in the group where the buccal bone is
216 more than 1 mm, which leaves a probability of 63.61% (458/720) for the group of buccal bone
217 that has less than 1 mm. In this group, there is a high risk for recession of the facial mucosa
218 and significant flattening the soft tissue profile at the neck of implant prosthesis [24]. It is
219 important to emphasize that the use of buccal bone augmentation [25], soft tissue graft [26],
220 the combination of bone augmentation and soft tissue graft or alternative option such as partial
221 tooth extraction therapy [27] can contribute to improve the overall aesthetic result, and
222 compensate with buccal contour remodeling in IIP.
223 Sagittal root position (SRP) could directly influence the position of immediate implant
224 placement. In the study of Kan JY et al. [13], the author suggested that class I SRP was
225 favorable and class IV SRP was regarded as a contraindication for immediate implant
226 placement. In this study, the distribution with probability results of class I and class IV SRP
227 were in concordance with the study of Kan JY, the highest distribution and probability is class
228 I 87.42% (633/720) with probability of 84.20% (533/633) and class IV 8.89% (64/720) with
229 the lowest probability of 0% (0/64). Surprisingly, the probability in class II is more than in
230 class III, 70% (14/20) and 33.33% (1/3) respectively, which were different from previous study.
231 These may due to sufficient apical bone to engage in traditional cement retained implant
232 prosthesis (incisal edge) in class II, whereas in class III inadequate apical bone causing implant
233 to extended outward of alveolar housing.
234 Direction of implant prosthesis can influence the position of IIP as well. Cement
235 retained implant prosthesis has more advantages for esthetics, such as absence of screw access
236 hole. In addition, long axis of implant coincides incisal edge, which creates a proper emergence
237 angle that similar to the natural teeth approximately 15° [17]. While a screw retained implant
238 prosthesis is beneficial for retrievability, reducing complication from the left-over cement,
239 which can further cause inflammation and bone loss [19, 28, 29]. However, the disadvantages
240 such as screw access hole might be esthetically compromised. Therefore, in the esthetic zone,
241 the implant should be placed at cingulum area. However, the more palatal the implant
242 placement, the more emergence angle and therefore makes a greater cervical contour [30]. The
243 mentioned problem caused peri-implantitis and recession on labial surface [31]. Angulated
244 screw channel avoids screw access hole to be placed in palatal area without compromised
245 esthetic [20]. In addition, the position of implant that used of angulated screw channel is on
246 labial aspect, which creates proper emergence angle. The results of this study revealed that if
247 using traditional prosthetic screw with 0-degree compensation the prosthesis is limited to
248 73.47% (529/720) cement retained with 2.64% (19/720) traditional screw retained prosthesis.
249 By incorporating angulated screw with 25-degree compensation could increase the probability
250 of screw retained prosthesis to 55.42% (399/720). In individual tooth, an angulated screw
251 channel could help increase the probability for screw retained implant prosthesis in central
252 incisor from 7.92% (19/240) to 72.08% (173/240), in lateral incisors from 0% to 50%
253 (120/240), and in canine from 0% to 47.92% (115/240). Nevertheless, it’s important to note
254 that an angle over 25° above limitation of angulated screw channel, IIP can be performed in
255 esthetic zone in cement retained implant prosthesis, which often in the direction of middle of
256 individual tooth, hence referred to “middle half” only, with an 11.11% (80/720) probability.
257 When considering in implant length, IIP required the engagement of 4 to 5 mm of bone
258 apical to tooth socket [9], this result in increased implant length than the traditional implant
259 placement. An implant length of 14 mm is the most frequently used in central incisor and lateral
260 incisor 80% (192/240) and 56.67% (136/240) respectively. An implant length of 16 mm is the
261 most frequently used for canine teeth 58.75% (141/240), as canine has the longest root length
262 of 15.83 ± 1.49 mm [32]. Beyond the mentioned implant length could result in over extrusion
263 of implant. When considering implant diameter, a diameter of 3.3 mm is the most frequently
264 used for central incisors 81.67% (196/240). A diameter of 2.9 mm is the most frequently used
265 in lateral incisor 57.92% (139/240), due to the least faciopalatal root dimension. For canine
266 teeth with widest faciopalatal 6.67 mm [33], a diameter of 3.5 mm is the most frequently used
267 77.92% (187/240). Noteworthy in our study, the diameter of 2.9 mm is used in lateral incisor
268 only, due to the manufacturer’s recommendation. Previous study had shown that narrow
269 diameter implant with < 3 mm has decrease survival rate [34] with increases the risk of implant
270 fracture, abutment and screw failure [35, 36]. Even though, when simulating 2.9 mm diameter
271 in central incisors and canines can increase the probability from 82.50% (198/240 teeth) to
272 87.50% (210/240) and 81.25% (195/240) to 82.08% (197/240).
273 In recent years, there are two very similar studies Erin et al. [37], and
274 Kan et al. [38]. In Erin [37] study was virtual implant planning in screw
275 retained implant direction using straight or angled screw channel abutment with different
276 angulation (5°, 10°, 15°). The process is not IIP and the implant diameter for each site was fix
277 based on standards for each tooth type (canine and central incisor 4.3 mm, lateral incisor 3.5
278 mm). The implant length was 10 mm for all implant. In Kan JY [38] study investigate the
279 probability of using straight screw-channel screw-retained restoration for IIP in maxillary
280 anterior teeth. The method was 2D implant planning using digital template. The digital template
281 for 3.5 mm diameter was used in central incisor and lateral incisor. The digital template for 4.3
282 mm diameter was used in central incisor and canine. The implant length was 13, 15 and 18
283 mm.
284 This study was virtual implant planning by to determine the probability of IIP in
285 esthetic zone. The directions of implant that added for more than the two mentioned studies
286 were cement retained implant prosthesis, angulated screw channel at the 25° angulation and
287 the middle of individual tooth. Various size of diameter (2.9, 3.3, 3.5, 3.75, 4.0, 4.5, 5 mm) and
288 length (6, 8, 10, 12, 14, 16, 18 mm) were included in our study to promote adaptability and
289 suitability in IIP in esthetic zone.
290 For limitation of our study to standardized the method, implant planning is limited to
291 static predetermined angulation to anatomical relation and implant size. This could limit
292 the probability as clinician can increase the depth of implant placement, modified implant
293 angulation within restorable position. Future study is suggested to study the effect of
294 probability associated with dynamic implant planning.
295 **Conclusion**
296 Within the limitation of this study, screw retained implant prosthesis allows for the
297 lowest probability 2.64% (19/720) to perform IIP in esthetic zone, while angulated screw is
298 the most probability 55.42% (399/720). Moreover, the use of angulated screw channel is
299 essential to increase the probability of screw retained implant prosthesis. However, the limit
300 of angulated screw could not compensate for all IIP position. A
301 careful individual site planning is advised to maximize treatment outcome.

302

303 **Reference**
304 1.	Kan JY, Rungcharassaeng K. Site development for anterior single implant esthetics:
305 the dentulous site. Compend Contin Educ Dent. 2001 Mar;22(3):221-6, 228, 230-1; quiz 32.
306 2.	Phillips K, Kois JC. Aesthetic peri-implant site development. The restorative
307 connection. Dent Clin North Am. 1998 Jan;42(1):57-70.
308 3.	Araujo MG, Lindhe J. Dimensional ridge alterations following tooth extraction. An
309 experimental study in the dog. J Clin Periodontol. 2005 Feb;32(2):212-8. Available from:
310 <https://doi.org/10.1111/j.1600-051x.2005.00642.x>
311 4.	Araújo MG, Wennström JL, Lindhe J. Modeling of the buccal and lingual bone walls
312 of fresh extraction sites following implant installation. Clin Oral Implants Res. 2006
313 Dec;17(6):606-14. Available from: <https://doi.org/10.1111/j.1600-0501.2006.01315.x>
314 5.	Sanz M, Cecchinato D, Ferrus J, Pjetursson EB, Lang NP, Lindhe J. A prospective,
315 randomized-controlled clinical trial to evaluate bone preservation using implants with
316 different geometry placed into extraction sockets in the maxilla. Clin Oral Implants Res. 2010
317 Jan;21(1):13-21. Available from: <https://doi.org/10.1111/j.1600-0501.2009.01824.x>
318 6.	Tomasi C, Sanz M, Cecchinato D, Pjetursson B, Ferrus J, Lang NP, et al. Bone
319 dimensional variations at implants placed in fresh extraction sockets: a multilevel
320 multivariate analysis. Clin Oral Implants Res. 2010 Jan;21(1):30-6. Available from:
321 <https://doi.org/10.1111/j.1600-0501.2009.01848.x>
322 7.	Huynh-Ba G, Pjetursson BE, Sanz M, Cecchinato D, Ferrus J, Lindhe J, et al.
323 Analysis of the socket bone wall dimensions in the upper maxilla in relation to immediate
324 implant placement. Clin Oral Implants Res. 2010 Jan;21(1):37-42. Available from:
325 <https://doi.org/10.1111/j.1600-0501.2009.01870.x>
326 8.	Spray JR, Black CG, Morris HF, Ochi S. The influence of bone thickness on facial
327 marginal bone response: stage 1 placement through stage 2 uncovering. Ann Periodontol.
328 2000 Dec;5(1):119-28. Available from: <https://doi.org/10.1902/annals.2000.5.1.119>
329 9.	Kan JY, Rungcharassaeng K. Immediate placement and provisionalization of
330 maxillary anterior single implants: a surgical and prosthodontic rationale. Pract Periodontics
331 Aesthet Dent. 2000 Nov-Dec;12(9):817-24; quiz 26.
332 10.	Levine RA, Ganeles J, Gonzaga L, Kan JK, Randel H, Evans CD, et al. 10 Keys for
333 Successful Esthetic-Zone Single Immediate Implants. Compend Contin Educ Dent. 2017
334 Apr;38(4):248-260.
335 11.	Rosa AC, da Rosa JC, Dias Pereira LA, Francischone CE, Sotto-Maior BS.
336 Guidelines for Selecting the Implant Diameter During Immediate Implant Placement of a
337 Fresh Extraction Socket: A Case Series. Int J Periodontics Restorative Dent. 2016 May-
338 Jun;36(3):401-7. Available from: <https://doi.org/10.11607/prd.2381>
339 12.	Theisen FC, Shultz RE, Elledge DA. Displacement of a root form implant into the
340 mandibular canal. Oral Surg Oral Med Oral Pathol. 1990 Jul;70(1):24-8. Available from:
341 <https://doi.org/10.1016/0030-4220(90)90172-o>
342 13.	Kan JY, Roe P, Rungcharassaeng K, Patel RD, Waki T, Lozada JL, et al.
343 Classification of sagittal root position in relation to the anterior maxillary osseous housing for
344 immediate implant placement: a cone beam computed tomography study. Int J Oral
345 Maxillofac Implants. 2011 Jul-Aug;26(4):873-6.
346 14.	Ganz SD. Presurgical planning with CT-derived fabrication of surgical guides. J Oral
347 Maxillofac Surg. 2005 Sep;63(9 Suppl 2):59-71. Available from:
348 <https://doi.org/10.1016/j.joms.2005.05.156>
349 15.	Fortin T, Champleboux G, Lormée J, Coudert JL. Precise dental implant placement in
350 bone using surgical guides in conjunction with medical imaging techniques. J Oral Implantol.
351 2000;26(4):300-3. Available from: https://doi.org/10.1563/1548-
352 1336(2000)026%3C0300:pdipib%3E2.3.co;2
353 16.	Hebel KS, Gajjar RC. Cement-retained versus screw-retained implant restorations:
354 achieving optimal occlusion and esthetics in implant dentistry. J Prosthet Dent. 1997
355 Jan;77(1):28-35. Available from: <https://doi.org/10.1016/s0022-3913(97)70203-8>
356 17.	Du JK, Li HY, Wu JH, Lee HE, Wang CH. Emergence angles of the cementoenamel
357 junction in natural maxillary anterior teeth. J Esthet Restor Dent. 2011 Dec;23(6):362-9.
358 Available from: <https://doi.org/10.1111/j.1708-8240.2011.00471.x>
359 18.	Linkevicius T, Vindasiute E, Puisys A, Peciuliene V. The influence of margin
360 location on the amount of undetected cement excess after delivery of cement-retained implant
361 restorations. Clin Oral Implants Res. 2011 Dec;22(12):1379-84. Available from:
362 <https://doi.org/10.1111/j.1600-0501.2010.02119.x>
363 19.	Michalakis KX, Hirayama H, Garefis PD. Cement-retained versus screw-retained
364 implant restorations: a critical review. Int J Oral Maxillofac Implants. 2003 Sep-
365 Oct;18(5):719-28.
366 20.	Garcia-Gazaui S, Razzoog M, Sierraalta M, Saglik B. Fabrication of a screw-retained
367 restoration avoiding the facial access hole: a clinical report. J Prosthet Dent. 2015
368 Nov;114(5):621-4. Available from: <https://doi.org/10.1016/j.prosdent.2015.06.007>
369 21.	Shreya Svitlana Anand, Ashok Velayudhan, Jaiganesh Ramamurthy. Immediate
370 Implant Placement in Anterior Maxilla – A Retrospective Study. Int. J. Res. Pharm. Sci.
371 .2020 Oct. 16 ;11(3):1452-7. Available from: <https://ijrps.com/home/article/view/3098>
372 22.	Cooper LF. Objective criteria: guiding and evaluating dental implant esthetics. J
373 Esthet Restor Dent. 2008;20(3):195-205. Available from: [https://doi.org/10.1111/j.1708-](https://doi.org/10.1111/j.1708-8240.2008.00178.x)
374 [8240.2008.00178.x](https://doi.org/10.1111/j.1708-8240.2008.00178.x)
375 23.	Januário AL, Duarte WR, Barriviera M, Mesti JC, Araújo MG, Lindhe J. Dimension
376 of the facial bone wall in the anterior maxilla: a cone-beam computed tomography study. Clin
377 Oral Implants Res. 2011 Oct;22(10):1168-1171. Available from:
378 <https://doi.org/10.1111/j.1600-0501.2010.02086.x>
379 24.	Buser D, Chappuis V, Belser UC, Chen S. Implant placement post extraction in
380 esthetic single tooth sites: when immediate, when early, when late? Periodontol 2000. 2017
381 Feb;73(1):84-102. Available from: <https://doi.org/10.1111/prd.12170>
382 25.	Chen K, Li Z, Liu X, Liu Q, Chen Z, Sun Y, Chen Z, Huang B. Immediate Implant
383 Placement with Buccal Bone Augmentation in the Anterior Maxilla with Thin Buccal Plate:
384 A One-Year Follow-Up Case Series. J Prosthodont. 2021 Jul;30(6):473-480. Available from:
385 <https://doi.org/10.1111/jopr.13350>
386 26.	El-Askary AS. Use of connective tissue grafts to enhance the esthetic outcome of
387 implant treatment: a clinical report of 2 patients. J Prosthet Dent. 2002 Feb;87(2):129-32.
388 Available from: <https://doi.org/10.1067/mpr.2002.121405>
389 27.	Hürzeler MB, Zuhr O, Schupbach P, Rebele SF, Emmanouilidis N, Fickl S. The
390 socket-shield technique: a proof-of-principle report. J Clin Periodontol. 2010 Sep;37(9):855-
391 62. Available from: <https://doi.org/10.1111/j.1600-051x.2010.01595.x>
392 28.	Vindasiute E, Puisys A, Maslova N, Linkeviciene L, Peciuliene V, Linkevicius T.
393 Clinical Factors Influencing Removal of the Cement Excess in Implant-Supported
394 Restorations. Clin Implant Dent Relat Res. 2015 Aug;17(4):771-8. Available from:
395 <https://doi.org/10.1111/cid.12170>
396 29.	Pauletto N, Lahiffe BJ, Walton JN. Complications associated with excess cement
397 around crowns on osseointegrated implants: a clinical report. Int J Oral Maxillofac Implants.
398 1999 Nov-Dec;14(6):865-8.
399 30.	Testori T, Weinstein T, Scutellà F, Wang HL, Zucchelli G. Implant placement in the
400 esthetic area: criteria for positioning single and multiple implants. Periodontol 2000. 2018
401 Jun;77(1):176-196. Available from: <https://doi.org/10.1111/prd.12211>
402 31.	Buser D, Martin W, Belser UC. Optimizing esthetics for implant restorations in the
403 anterior maxilla: anatomic and surgical considerations. Int J Oral Maxillofac Implants.
404 2004;19 Suppl:43-61.
405 32.	Kim SY, Lim SH, Gang SN, Kim HJ. Crown and root lengths of incisors, canines,
406 and premolars measured by cone-beam computed tomography in patients with malocclusions.
407 Korean J Orthod. 2013 Dec;43(6):271-8. Available from:
408 <https://doi.org/10.4041/kjod.2013.43.6.271>
409 33.	Lee SL, Kim HJ, Son MK, Chung CH. Anthropometric analysis of maxillary anterior
410 buccal bone Korean adults using cone-beam CT. J Adv Prosthodont. 2010 Sep;2(3):92-6.
411 Available from: <https://doi.org/10.4047/jap.2010.2.3.92>
412 34.	Schiegnitz E, Al-Nawas B. Narrow-diameter implants: A systematic review and meta-
413 analysis. Clin Oral Implants Res. 2018 Oct;29 Suppl 16:21-40. Available from:
414 <https://doi.org/10.1111/clr.13272>
415 35.	Petrie CS, Williams JL. Comparative evaluation of implant designs: influence of
416 diameter, length, and taper on strains in the alveolar crest. A three-dimensional finite-element
417 analysis. Clin Oral Implants Res. 2005 Aug;16(4):486-94. Available from:
418 <https://doi.org/10.1111/j.1600-0501.2005.01132.x>
419 36.	Quek CE, Tan KB, Nicholls JI. Load fatigue performance of a single-tooth implant
420 abutment system: effect of diameter. Int J Oral Maxillofac Implants. 2006; Nov-
421 Dec21(6):929-36.
422 37.	Erin K Edmondson, Pedro M Trejo, Nikolaos Soldatos, Robin L Weltman. The ability
423 to screw-retain single implant-supported restorations in the anterior maxilla: A CBCT
424 analysis. J Prosthet Dent. 2022 Sep;128(3):443-449. Available from:
425 <https://doi.org/10.1016/j.prosdent.2021.01.004>
426 38.	Joseph Y K Kan, Kitichai Rungcharassaeng, Pongrapee Kamolroongwarakul, Guo-
427 Hao Lin, Hiroyuki Matsuda, Shi Yin, Hom-Lay Wang, Dennis Tarnow, Jaime L Lozada.
428 Frequency of screw-retained angulated screw channel single crown following immediate
429 implant placement and provisionalization in the esthetic zone: A cone beam computed
430 tomography study/ Clin Implant Dent Relat Res. 2023 Oct;25(5):789-794. Available from:
431 <https://doi.org/10.1111/cid.13227>

<https://www.editorialmanager.com/pone/download.aspx?id=35905294&guid=f33f1339-b75a-4b3b-92ff-99a132c6dea3&scheme=1>Figure1	[Click here to access/download;Figure;Fig 1.tif](https://www.editorialmanager.com/pone/download.aspx?id=35905294&guid=f33f1339-b75a-4b3b-92ff-99a132c6dea3&scheme=1)

<https://www.editorialmanager.com/pone/download.aspx?id=35905295&guid=7c265615-dc6c-471e-9afe-8ebb88358433&scheme=1>Figure2	[Click here to access/download;Figure;Fig 2.tif](https://www.editorialmanager.com/pone/download.aspx?id=35905295&guid=7c265615-dc6c-471e-9afe-8ebb88358433&scheme=1)

<https://www.editorialmanager.com/pone/download.aspx?id=35905297&guid=2d4abe4a-b393-4f38-b042-3748aa05a163&scheme=1>Figure3	[Click here to access/download;Figure;Fig 3.tif](https://www.editorialmanager.com/pone/download.aspx?id=35905297&guid=2d4abe4a-b393-4f38-b042-3748aa05a163&scheme=1)

Supporting Information

# [Click here to access/download](https://www.editorialmanager.com/pone/download.aspx?id=35905287&guid=5c087df2-3e40-4ba6-a66c-6eb29c002ad3&scheme=1)

[Supporting Information Supporting information.xlsx](https://www.editorialmanager.com/pone/download.aspx?id=35905287&guid=5c087df2-3e40-4ba6-a66c-6eb29c002ad3&scheme=1)

Revised Manuscript with Track Changes

1

**Incidence of straight and angulated screw channel following immediate implant placement in esthetic zone: a CBCT study**

Watcharapon Radomsutthisarn, DDS 1¶, Pranai Nakaparksin, DDS, MSD, FACP, FRCD(c)

1&*, #a, Kornkamol Kretapirom DDS, PhD ^2&^

1 Department of Advanced General Dentistry, Faculty of Dentistry, Mahidol University, Bangkok, Thailand,
2 Department of Oral and Maxillofacial Radiology, Faculty of Dentistry, Mahidol University,

Bangkok, Thailand

#a Current address: Department of Advanced General Dentistry, Faculty of Dentistry, Mahidol University, Bangkok, Thailand

**Corresponding Authors:**

Email: [Pranai.nak@mahidol.ac.th](mailto:Pranai.nak@mahidol.ac.th)

¶These authors contributed equally to this work. &These authors also contributed equally to this work.

1

2 **Abstract**
3 **Statement of problem:** What are the incidences of immediate implant placement (IIP) in the
4 esthetic zone, using four different implant directions?
5 **Purpose:** The purpose of this cone-beam computed tomography (CBCT) study was to
6 determine the probability of IIP in the esthetic zone, using four different implant directions.
7 **Materials and methods:** A retrospective review of CBCT images was conducted as a part of
8 this study and accessed for research purpose on 9 June 2021. The midsagittal images of
9 maxillary anterior teeth (right canine to left canine) were input into an implant planning
10 software (Implant Studio®). Bone Level X Straumann® implant (BLX 3.5, 3.75, 4.0, 4.5, 5.0
11 mm) and Bone Level Tapered implant (BLT 2.9, 3.3 mm) were selected for this study. The

**Formatted:** Font: (Default) Times New Roman

12 diameter of 2.9 mm was used in lateral incisor only. The frequency distribution and

**Formatted:** Font: (Default) Times New Roman

13 probability of IIP were recorded and compared among all maxillary anterior teeth.
14 **Results:** CBCT images from 720 teeth (120 patient) were evaluated. We found the overall
15 probability of IIP in the esthetic zone is 76.11% (548/720). Four different implant directions
16 were evaluated in this study, with the following results; traditional screw retained implant
17 prosthesis (screw retained), traditional cement retained implant prosthesis (incisal edge),
18 limited angulation for angulated screw channel (angle screw) and limited restorability
19 angulation for cement retained implant prosthesis (middle half) are 2.64% (19/720), 26.11%
20 (188/720), 55.42% (399/720) and 38.75% (279/720) respectively.
21 **Conclusion:** Angulated screw channels is imperative in increasing the probability of IIP for
22 screw retained implant prosthesis.
23 **Clinical implications:** For communication with the patient to determine treatment options
24 for replacement of failing teeth in esthetic zone. To understand how that angulated screw
25 channel can increase the chance for IIP in the dental esthetic zone.
26 **Introduction**
27 Immediate implant placement (IIP) following tooth extraction has become a common
28 clinical procedure for the replacement of failing teeth. However, placement of dental implants
29 in the maxillary anterior esthetic zone is challenging for clinicians, due to the patients’ esthetic
30 expectations and several risk factors that affect overall outcome of the treatment. Therefore,
31 for desired esthetic results and tooth function when performing an IIP [1, 2], this procedure
32 requires adequate information (clinical examination, radiographic examination, cone beam
33 computed tomography (CBCT)).
34 Previous studies indicated that the morphological bone changes immediately following
35 tooth extraction. As a result, the alveolar bone and subsequent implant placement can be
36 affected [3-6]. These human studies measured the width of the buccal bone walls of the
37 extraction sockets in the esthetic zone and determined that the mean width of the buccal bony
38 wall in maxillary anterior teeth was 1mm. The majority of the buccal bony walls (87.2%) had
39 a width ≤ 1 mm, while only 2.6% of the walls measured 2 mm wide [7]. In addition, in the
40 study of Spray et al. [8] measured the changes in the vertical bone at the time of implant
41 insertion and compared these changes to the thickness of the facial bones. The findings of this
42 study revealed that when the facial bone reaches a thickness of 1.8 mm to 2 mm, bone loss
43 significantly decreased. The results showed that the optimal “critical thickness” value for
44 minimizing implant issues and facial bone loss in the facial plate, was approximately 2 mm [8].
45 In these studies, the buccal bone wall in maxillary anterior teeth was 1 mm, and most of them
46 had a width of ≤ 1 mm. Therefore, the recommended prerequisite for bone graft material is a
47 minimum of 2 mm distance between the implant and the internal part of the buccal bone wall.
48 This should be enough to limit midfacial mucosal recession in the future [9].
49 It is important to accurately determine buccal bone thickness, palatal bone thickness,
50 and faciopalatal root diameter to select the appropriate implant diameter and length, in order to
51 prevent unfavorable esthetic consequences following morphological bone changes in the facial
52 aspect. For example, when placing a larger diameter implant, the vascular supply to the buccal
53 plate is compromised, which increases the risk of resorption and recession particularly in the
54 midfacial region. However, when using a smaller diameter implant this can result in the tooth
55 having lower primary stability [10, 11]. When considering which implant length to use, if the
56 implant length is too long, it will invade critical structures such as the inferior alveolar nerve
57 or maxillary sinus, thus generating more trauma. If implant length is too short, it will reduce
58 osteointegration into the alveolar bone, thus reducing in primary stability [12]. Because IIP
59 requires bone engagement at apical area 4-5 mm beyond the tooth socket apex [9], implant
60 length should be considered preoperatively.
61 A preoperative CBCT scan provides valuable information about the site when placing
62 implants in the esthetic zone, including the current buccal and palatal plate width, expected
63 implant diameter and length, and sagittal root position (SRP) [13-15]. Therefore, a preoperative
64 CBCT scan is highly recommended and provides much needed advanced radiographs for
65 proper treatment planning of IIP in the esthetic zone.
66 Restoration on implant can be either screw retained restoration or cement retained
67 restoration [16]. However, cement retained restoration have more advantage in esthetics since
68 they can provide more suitable emergence angle [17] without showing screw access hole. So,
69 the cement retained restoration are better options for concealing the access hole and practical
70 to use in esthetic zone. Nevertheless, the main drawback of the cement retained restoration is
71 difficulty in cleaning excess cement when performing subgingival margin [18]. While, in
72 screw retained was good in retrievability and not presented excess cement [19]. However,
73 screw access hole will be compromised esthetic. Where the screw access is visible, screw
74 retained can’t be an option. Cement retained restorations are favorable because they allow for
75 more implant positions [16]. Angulated screw channel allows the screw access hole to be
76 placed with an angle up to 25° from long axis of implant. Thus, using an angulated screw
77 channel to avoid the screw access hole, allows fabricating the screw retained restoration
78 without compromise esthetic results [20].
79 The purpose of this study was to determine the probability of IIP in the esthetic zone,
80 using four different implant directions, traditional screw retained implant prosthesis (screw
81 retained), traditional cement retained implant prosthesis (incisal edge), limited angulation for
82 angulated screw channel (angle screw), and limit restorability angulation for cement retained
83 implant prosthesis (middle half).
84 **Material and method**
85 **Patient selection**
86 This retrospective study was approved by the Institutional Review Board (IRB) of the
87 Faculty of Dentistry/Faculty of Pharmacy, Mahidol University (IRB 2021/052.0906). The
88 sample criteria for this study were patients who had CBCT scans of the anterior maxilla at the
89 Oral and Maxillofacial Radiology clinic, Faculty of Dentistry, Mahidol University performed
90 during January 2012 – August 2022. CBCT images were performed by 3D Accuitomo 170 (J.
91 Morita®, Japan) and randomly selected for evaluation on 9 June 2021. The sample size
92 calculation for estimating proportion will be used with ~~the related~~retrospective study of Asok

**Formatted:** Not Highlight

93 Velayudhan et al [21], which the objective related to our study ~~[21]~~. The sample size calculation
94 is 120 CBCT scans according to the following criteria.
95 Inclusion criteria were as follows:
96 - CBCT must have field of view (FOV) 6 cm. x 6 cm. (voxel size 0.125 mm) or FOV 8 cm. x
97 8 cm. (voxel size 0.160 mm.).
98 - The patient must be at least 18 years old at the time of CBCT scan.
99 - The patient must have all maxillary anterior teeth (canine to canine).
100 Exclusion criteria were as follows:
101 **-** Radiographic evidence of periapical inflammatory lesion, periodontitis, severe root
102 resorption, trauma, or bone pathology.
103 - Radiographic evidence of artifacts and orthodontic appliances.
104 - Upper anterior teeth were misaligned.
105 **Data reconstruction and CBCT image**
106 CBCT volumetric data was exported to DICOM files and reconstructed by using
107 Implant Studio® (3Shape®, Denmark). For the axial view, the implant was positioned at the
108 center of the selected tooth. In the panoramic view, the implant was aligned parallel to the
109 long axis of the tooth. In the coronal view, the implant was rotated to be parallel to the long
110 axis of the tooth. The mid-sagittal image, which is located at the bucco-lingual side of the
111 tooth, was used to measure all parameters (Fig 1).
112 **Fig 1 User interface of Implant Studio® software showing the implant placement at**
113 **area 13**.
114 The mid-sagittal of the selected tooth was used for the measurement of the buccal bone
115 thickness, palatal bone thickness, and faciopalatal root dimension at the bone crest level. SRP
116 was classified with respect to the alveolar housing according to Kan JY et al. [13].
117 **Implant planning and determining of implant diameter and length**
118 Implant was planned by Implant Studio® (3Shape®, Denmark) using Bone level X
119 Straumann® implant (BLX 3.5, 3.75, 4.0, 4.5, 5.0 mm) (Straumann®, Switzerland) and Bone
120 Level Tapered implant (BLT 2.9, 3.3 mm) (Straumann®, Switzerland). BLT 2.9 mm was used
121 in lateral incisor only. The angulated screw channel was included in the calculation of all

**Formatted:** Not Highlight

122 implant diameter except diameter of 2.9 mm was not included.
123 To achieve restorative driven, an implant should be placed 3 mm apical and 2 mm
124 palatal to gingival margin [22]. Thus, the 3 mm line apical to gingival margin and a 2 mm line,
125 which perpendicular to the first line were created. The end point was marked as a position of
126 the most buccal aspect of implant platform (point P). The line drawn from incisal edge to root

**Formatted:** Not Highlight

127 apex was long axis of the tooth. The point between incisal edge to CEJ was marked as point
128 M. The point between the center of the most prominent point of cingulum to the incisal edge
129 was marked as point S. In traditional cement retained implant prosthesis (incisal edge), long
130 axis of implant will coincide with incisal edge. In traditional screw retained implant prosthesis
131 (screw retained), long axis of implant will coincide with point S. In limit angulation for
132 angulated screw channel (angle screw), long axis of implant will coincide at the point that an
133 angle up to 25° from traditional screw retained. In limit restorability angulation for cement
134 retained implant prosthesis (middle half), long axis of implant will coincide with point M ~~The~~
135 ~~distance between the center of the most prominent point of cingulum to the incisal edge was~~
136 ~~marked as point S. The middle of an individual tooth was marked as point M~~. The implant
137 component points and lines are shown in Fig 2.
138 **Fig 2 The component points of the implant are marked in mid-sagittal images.**
139 The implant was positioned in four different directions (Fig 3) as follows: screw
140 retained, ~~(long axis of implant will coincide with point S),~~ incisal edge, ~~(long axis of implant~~
141 ~~will coincide with incisal edge)~~, angle screw and ~~screw (long axis of implant will coincide at~~
142 ~~the point that an angle up to 25° from long axis of implant),~~ middle half. ~~(long axis of implant~~
143 ~~will coincide with the point M).~~
144 **Fig 3 The four different implant directions in this study.**
145 To determine the implant diameter and length, the smallest implant diameter and length
146 were selected. The implant length was increased until engaging a minimum of 4 mm in distance
147 from the apical to root apex [9], which is considered as the first implant position and increased
148 until the apex of implant is positioned 1 mm away from critical structures. After that, increased
149 the next size implant diameter until it reached the maximum, which calculated by faciopalatal
150 root dimension minus two and repeated protocol for the implant length.
151 All measurements (the buccal bone thickness, palatal bone thickness, and faciopalatal
152 root dimension) for the implant planning process were performed by one examiner. The
153 intraexaminer reliability was assessed by repeating the measurement on 10 randomly selected
154 CBCT images 1 month after the first measurement by intra-class correlation coefficient (ICC).
155 Descriptive statistics were used to report the probability of IIP in the esthetic zone
156 using four different implant directions as percentages.
157 **Results**
158 120 CBCT images from maxillary anterior teeth (240 central incisors, 240 lateral
159 incisors, 240 canines) in 120 patients (39 males and 81 females) with a mean age of 35 years
160 old (range: 21 to 77 years old) were evaluated in this study. The ICC of this study is 0.885
161 (good reliability).
162 The result revealed that 84.58 % (609/720) of anterior maxillary teeth had buccal bone
163 of less than 1 mm. The SRP with probability of IIP are as follow: class I 87.91% (633/720)
164 with 84.20% (533/633), class II 2.78% (20/720) with 70% (14/20), class III 0.42% (3/720) with
165 33.33% (1/3) and class IV 8.89% (64/720) with 0% respectively.
166 The distribution of SRP with probability of IIP are shown in Table 1.
167 The probability of IIP in all maxillary anterior teeth are 76.11% (548/720), with consist
168 of central incisor 82.50% (198/240), lateral incisor 64.58% (155/240) and canine 81.25%
169 (195/240). In this study, an individual tooth can have multiple implant positions. The
170 probability of IIP for each relation (10 relations) is shown in Table 2.
171 The probability of IIP in screw retained, incisal edge, angle screw and middle half are

172	2.64% (19/720), 26.11% (188/720), 55.42% (399/720) and 38.75% (279/720) respectively.

173 The probability of IIP in each direction is shown in Table 3.
174 Central incisor can position implant with diameter of 3.3 mm, 3.5 mm, 3.75 mm, 4.0

175	mm and 4.5 mm at 81.67% (196/240), 68.33% (164/240), 54.58% (131/240), 40.83% (98/240)

176 and 5% (12/240) respectively and length of 10 mm, 12 mm, 14 mm, 16 mm and 18 mm at
177 22.5% (54/240), 62.92% (151/240), 80% (192/240), 73.33% (176/240) and 54.58% (131/240)
178 respectively.
179 Lateral incisor can position implant with diameter of 2.9 mm, 3.3 mm, 3.5 mm, 3.75

180	mm and 4.0 mm at 57.92% (139/240), 51.25% (123/240), 36.25% (87/240), 26.67% (64/240)

181	and 15% (36/240) respectively and length of 10 mm, 12 mm, 14 mm, 16 mm and 18 mm at

| 182 | 6.67% (16/240), 30% (72/240), 56.67% (136/240), 43.33% (104/240) and 30.83% (74/240) |
| --- | --- |
| 183 | respectively. |
| 184 | Canine can position implant with diameter of 3.3 mm, 3.5 mm, 3.75 mm, 4.0 mm, 4.5 |
| 185 | mm and 5.0 mm at 73.33% (176/240), 77.92% (187/240), 75.42% (181/240), 71.25% |
| 186 | (171/240), 55% (132/240) and 23.75% (57/240) respectively and length of 10 mm, 12 mm, 14 |
| 187 | mm, 16 mm and 18 mm at 7.5% (18/240), 25.42% (61/240), 49.58% (119/240), 58.75% |
| 188 | (141/240) and 55% (132/240) respectively. |
| 189 | The distribution and the percentages of the diameter and length, which were used for |
| 190 | IIP are shown in Table 4. |
| 191 |  |

| **Table 1 Distribution of sagittal root position** (**SRP**) **with probability of IIP in esthetic zone** | | | | | | | | | | | | |
| --- | --- | --- | --- | --- | --- | --- | --- | --- | --- | --- | --- | --- |
| **SRP** | **Class I** | | | **Class II** | | | **Class III** | | | **Class IV** | | |
| **Distribution** | **633**/**720** (**87.91%**) | | | **20**/**720** (**2.78%**) | | | **3**/**720** (**0.42%**) | | | **64**/**720** (**8.89%**) | | |
| **Probability** | **533**/**633** (**84.20%**) | | | **14**/**20** (**70%**) | | | **1**/**3** (**33.33%**) | | | **0**/**64** (**0%**) | | |
|  | **Central** | **Lateral** | **Canine** | **Central** | **Lateral** | **Canine** | **Central** | **Lateral** | **Canine** | **Central** | **Lateral** | **Canine** |
| **Distribution** | **213**(**88.75%**) | **183**(**76.25%**) | **237**(**98.75%**) | **9**(**3.75%**) | **11**(**4.58%**) | **-** | **1**(**0.42%**) | **2**(**0.83%**) | **-** | **17**(**7.08%**) | **44**(**18.34%**) | **3**(**1.25%**) |

192

193

194

| **Table 2 Detailed probability of IIP by angulation groups and tooth type** | | | | |
| --- | --- | --- | --- | --- |
|  | **Central incisor** (**240 teeth**) | **Lateral incisor** (**240 teeth**) | **Canine** (**240 teeth**) | **Total** (**720 teeth**) |
| **1. Incisal edge** (**I**) | **24**(**10%**) | **29**(**12.08%**) | **7**(**2.92%**) | **60**(**8.33%**) |
| **2. Screw retained** (**S**) | **1**(**0.42%**) | **-** | **-** | **1**(**0.14%**) |
| **3. Angle screw** (**A**) | **57**(**23.75%**) | **52**(**21.66%**) | **21**(**8.75%**) | **130**(**18.06%**) |
| **4. Middle half** (**M**) | **1**(**0.42%**) | **6**(**2.5%**) | **73**(**30.41%**) | **80**(**11.11%**) |
| **5. I, S** | **8**(**3.33%**) | **-** | **-** | **8**(**1.11%**) |
| **6. I, A** | **47**(**19.58%**) | **10**(**4.17%**) | **6**(**2.5%**) | **63**(**8.75%**) |
| **7. A, M** | **26**(**10.83%**) | **45**(**18.75%**) | **78**(**32.5%**) | **149**(**20.69%**) |
| **8. I, S, A** | **7**(**2.92%**) | **-** | **-** | **7**(**0.97%**) |
| **9. I, A, M** | **24**(**10%**) | **13**(**5.42%**) | **10**(**4.17%**) | **47**(**6.53%**) |
| **10. I, S, A, M** | **3**(**1.25%**) | **-** | **-** | **3**(**0.42%**) |
| **Total** | **198/240**(**82.5%**) | **155**/**240**(**64.58%**) | **195**(**81.25%**) |  |

195

196

197

198

199

| **Table 3 Summary of IIP probability by angulation groups and tooth type** | | | | |
| --- | --- | --- | --- | --- |
| **Angulation** | **Central incisor** (**240 teeth**) | **Lateral incisor** (**240 teeth**) | **Canine** (**240 teeth**) | **Total** (**720 teeth**) |
| **Incisal edge**  **1, 5, 6, 8, 9, 10** | **113**(**47.08%**) | **52**(**21.67%**) | **23**(**9.58%**) | **188**(**26.11%**) |
| **Screw retained**  **2, 5, 8, 10** | **19**(**7.92%**) | **0** | **0** | **19**(**2.64%**) |
| **Angle screw**  **3, 6, 7, 8, 9, 10** | **164**(**68.33%**) | **120**(**50%**) | **115**(**47.92%**) | **399**(**55.42%**) |
| **Middle half**  **4, 7, 9, 10** | **54**(**22.5%**) | **64**(**26.67%**) | **161**(**67.08%**) | **279**(**38.75%**) |

200

201

202 1. I = incisal edge, 2. S = screw, 3. A = angle, 4. M = middle, 5. I, S = incisal edge and screw, 6. I, A = incisal edge and angle, 7. A, M = angle
203 and middle, 8. I, S, A = incisal edge, screw and angle, 9. I, A, M = incisal edge, angle and middle, 10. I, S, A, M = incisal edge, screw, angle and
204 middle

| **Table 4 Distributions of diameter and length of implant used in immediate implant placement in esthetic zone** | | | | | | | | | | | | | |
| --- | --- | --- | --- | --- | --- | --- | --- | --- | --- | --- | --- | --- | --- |
| **Diameter** | | | | | | | **Length** | | | | | | |
|  | **Central**  (**240 teeth**) | | **Lateral**  (**240 teeth**) | | **Canine**  (**240 teeth**) | |  | **Central**  (**240 teeth**) | | **Lateral**  (**240 teeth**) | | **Canine**  (**240 teeth**) | |
| **2.9 mm** | **-** | **-** | **139** | **57.92%** | **-** | **-** | **6 mm** | **-** | **-** | **-** | **-** | **-** | **-** |
| **3.3 mm** | **196** | **81.67%** | **123** | **51.25%** | **176** | **73.33%** | **8 mm** | **-** | **-** | **-** | **-** | **-** | **-** |
| **3.5 mm** | **164** | **68.33%** | **87** | **36.25%** | **187** | **77.92%** | **10 mm** | **54** | **22.5%** | **16** | **6.67%** | **18** | **7.5%** |
| **3.75 mm** | **131** | **54.58%** | **64** | **26.67%** | **181** | **75.42%** | **12 mm** | **151** | **62.92%** | **72** | **30%** | **61** | **25.42%** |
| **4.0 mm** | **98** | **40.83%** | **36** | **15%** | **171** | **71.25%** | **14 mm** | **192** | **80%** | **136** | **56.67%** | **119** | **49.58%** |
| **4.5 mm** | **12** | **5%** | **-** | - | **132** | **55%** | **16 mm** | **176** | **73.33%** | **104** | **43.33%** | **141** | **58.75%** |
| **5 mm** | **-** | **-** | **-** | **-** | **57** | **23.75%** | **18 mm** | **131** | **54.58%** | **74** | **30.83%** | **132** | **55%** |

205

206

207

208

209

210 **Discussion**
211 The success criteria for immediate implant placement (IIP) in the esthetic zone
212 including non-acute infection areas, presence of thick gingival biotype, engaging sufficient
213 apical bone, with intact buccal bone [9]. Recent studies have shown that on average, the buccal
214 bone in anterior maxillary teeth was less than 1 mm [7, 23]. This study found similar results
215 with the majority of anterior maxillary teeth, 84.58% (609/720), having less than 1 mm of
216 buccal bone thickness. Canine teeth 29.17% (70/240), were found most frequently to have a
217 buccal bone thickness greater than 1 mm, followed by the lateral incisors and central incisors
218 11.25% (27/240) and 5.83% (14/240) respectively.
219 There was a 12.5% (90/720) probability of IIP in the group where the buccal bone is
220 more than 1 mm, which leaves a probability of 63.61% (458/720) for the group of buccal bone
221 that has less than 1 mm. In this group, there is a high risk for recession of the facial mucosa
222 and significant flattening the soft tissue profile at the neck of implant prosthesis [24]. It is
223 important to emphasize that the use of buccal bone augmentation [25], soft tissue graft [26],
224 the combination of bone augmentation and soft tissue graft or alternative option such as partial
225 tooth extraction therapy [27] can contribute to improve the overall aesthetic result, and
226 compensate with buccal contour remodeling in IIP.
227 Sagittal root position (SRP) could directly influence the position of immediate implant
228 placement. In the study of Kan JY et al. [13], the author suggested that class I SRP was
229 favorable and class IV SRP was regarded as a contraindication for immediate implant
230 placement. In this study, the distribution with probability results of class I and class IV SRP
231 were in concordance with the study of Kan JY, the highest distribution and probability is class
232 I 87.42% (633/720) with probability of 84.20% (533/633) and class IV 8.89% (64/720) with
233 the lowest probability of 0% (0/64). Surprisingly, the probability in class II is more than in class
234 III, 70% (14/20) and 33.33% (1/3) respectively, which were different from previous study.
235 These may due to sufficient apical bone to engage in traditional cement retained implant
236 prosthesis (incisal edge) in class II, whereas in class III inadequate apical bone causing implant
237 to extended outward of alveolar housing.
238 Direction of implant prosthesis can influence the position of IIP as well. Cement
239 retained implant prosthesis has more advantages for esthetics, such as absence of screw access
240 hole. In addition, long axis of implant coincides incisal edge, which creates a proper emergence
241 angle that similar to the natural teeth approximately 15° [17]. While a screw retained implant
242 prosthesis is beneficial for retrievability, reducing complication from the left-over cement,
243 which can further cause inflammation and bone loss [19, 28, 29]. However, the disadvantages
244 such as screw access hole might be esthetically compromised. Therefore, in the esthetic zone,
245 the implant should be placed at cingulum area. However, the more palatal the implant
246 placement, the more emergence angle and therefore makes a greater cervical contour [30]. The
247 mentioned problem caused peri-implantitis and recession on labial surface [31]. Angulated
248 screw channel avoids screw access hole to be placed in palatal area without compromised
249 esthetic [20]. In addition, the position of implant that used of angulated screw channel is on
250 labial aspect, which creates proper emergence angle. The results of this study revealed that if
251 using traditional prosthetic screw with 0-degree compensation the prosthesis is limited to
252 73.47% (529/720) cement retained with 2.64% (19/720) traditional screw retained prosthesis.
253 By incorporating angulated screw with 25-degree compensation could increase the probability
254 of screw retained prosthesis to 55.42% (399/720). In individual tooth, an angulated screw
255 channel could help increase the probability for screw retained implant prosthesis in central
256 incisor from 7.92% (19/240) to 72.08% (173/240), in lateral incisors from 0% to 50%
257 (120/240), and in canine from 0% to 47.92% (115/240). Nevertheless, it’s important to noted
258 that an angle over 25° above limitation of angulated screw channel, IIP can be performed in
259 esthetic zone in cement retained implant prosthesis, which often in the direction of middle of
260 individual tooth, hence referred to “middle half” ~~the direction of middle half~~ only, with an

**Formatted:** Not Highlight

261 11.11% (80/720) probability.
262 When considering in implant length, IIP required the engagement of 4 to 5 mm of bone apical
263 to tooth socket [9], this result in increased implant length than the traditional implant
264 placement. An implant length of 14 mm is the most frequently used in central incisor and lateral
265 incisor 80% (192/240) and 56.67% (136/240) respectively. An implant length of 16 mm is the
266 most frequently used for canine teeth 58.75% (141/240), as canine has the longest root length
267 of 15.83 ± 1.49 mm [32]. Beyond the mentioned implant length could result in over extrusion
268 of implant. When considering implant diameter, a diameter of 3.3 mm is the most frequently
269 used for central incisors 81.67% (196/240). A diameter of 2.9 mm is the most frequently used
270 in lateral incisor 57.92% (139/240), due to the least faciopalatal root dimension. For canine
271 teeth with widest faciopalatal 6.67 mm [33], a diameter of 3.5 mm is the most frequently used
272 77.92% (187/240). Noteworthy in our study, the diameter of 2.9 mm is used in lateral incisor
273 only, due to the manufacturer’s recommendation. Previous study had shown that narrow
274 diameter implant with < 3 mm has decrease survival rate [34] with increases the risk of implant
275 fracture, abutment and screw failure [35, 36]. Even though, when simulating 2.9 mm diameter
276 in central incisors and canines can increase the probability from 82.50% (198/240 teeth) to
277 87.50% (210/240) and 81.25% (195/240) to 82.08% (197/240).
278 In recent years, there are two very similar studies Erin K Edmondson et al. [37], and
279 Kan JY et al. [38]. In Erin K Edmondson [37] study was virtual implant planning in screw
280 retained implant direction using straight or angled screw channel abutment with different
281 angulation (5°, 10°, 15°). The process is not IIP and the implant diameter for each site was fix
282 based on standards for each tooth type (canine and central incisor 4.3 mm, lateral incisor 3.5
283 mm). The implant length was 10 mm for all implant. In Kan JY [38] study investigate the
284 probability of using straight screw-channel screw-retained restoration for IIP in maxillary
285 anterior teeth. The method was 2D implant planning using digital template. The digital template
286 for 3.5 mm diameter was used in central incisor and lateral incisor. The digital template for 4.3
287 mm diameter was used in central incisor and canine. The implant length was 13, 15 and 18
288 mm.
289 In our study was virtual implant planning by to determine the probability of IIP in
290 esthetic zone. The directions of implant that added for more than the two mentioned studies
291 were cement retained implant prosthesis, angulated screw channel at the 25° angulation and
292 the middle of individual tooth. Various size of diameter (2.9, 3.3, 3.5, 3.75, 4.0, 4.5, 5 mm) and
293 length (6, 8, 10, 12, 14, 16, 18 mm) were included in our study to promote adaptability and
294 suitability in IIP in esthetic zone.
295 For limitation of our study to standardized the method, implant planning is limited to
296 static predetermined angulation to anatomical relation and implant size. This could limited
297 the probability as clinician can increase the depth of implant placement, modified implant
298 angulation within restorable position. Future study is suggested to study the effect of
299 probability associated with dynamic implant planning.

300

301 **Conclusion**
302 Within the limitation of this study, screw retained implant prosthesis allows for the
303 lowest probability 2.64% (19/720) to perform IIP in esthetic zone, while angulated screw is
304 the most probability 55.42% (399/720). Moreover, the use of angulated screw channel is
305 essential to increase the probability of screw retained implant prosthesis. However, the limit
306 of angulated screw could not compensate for all immediate implant placement position. A
307 careful individual site planning is advised to maximize treatment outcome.

**Formatted:** Font: (Default) Times New Roman

**Formatted:** Font: (Default) Times New Roman

**Formatted:** Font: (Default) Times New Roman

**Formatted:** Font: (Default) Times New Roman

**Formatted:** Font: (Default) Times New Roman

**Formatted:** Font: (Default) Times New Roman

**Formatted:** Font: (Default) Times New Roman

**Formatted:** Font: (Default) Times New Roman

308

309

310 **Reference**
311 1.	Kan JY, Rungcharassaeng K. Site development for anterior single implant esthetics:
312 the dentulous site. Compend Contin Educ Dent. 2001 Mar;22(3):221-6, 228, 230-1; quiz 32.
313 2.	Phillips K, Kois JC. Aesthetic peri-implant site development. The restorative
314 connection. Dent Clin North Am. 1998 Jan;42(1):57-70.
315 3.	Araujo MG, Lindhe J. Dimensional ridge alterations following tooth extraction. An
316 experimental study in the dog. J Clin Periodontol. 2005 Feb;32(2):212-8. Available from:
317 <https://doi.org/10.1111/j.1600-051x.2005.00642.x>
318 4.	Araújo MG, Wennström JL, Lindhe J. Modeling of the buccal and lingual bone walls
319 of fresh extraction sites following implant installation. Clin Oral Implants Res. 2006
320 Dec;17(6):606-14. Available from: <https://doi.org/10.1111/j.1600-0501.2006.01315.x>
321 5.	Sanz M, Cecchinato D, Ferrus J, Pjetursson EB, Lang NP, Lindhe J. A prospective,
322 randomized-controlled clinical trial to evaluate bone preservation using implants with
323 different geometry placed into extraction sockets in the maxilla. Clin Oral Implants Res. 2010
324 Jan;21(1):13-21. Available from: <https://doi.org/10.1111/j.1600-0501.2009.01824.x>

325

326 6.	Tomasi C, Sanz M, Cecchinato D, Pjetursson B, Ferrus J, Lang NP, et al. Bone
327 dimensional variations at implants placed in fresh extraction sockets: a multilevel
328 multivariate analysis. Clin Oral Implants Res. 2010 Jan;21(1):30-6. Available from:
329 <https://doi.org/10.1111/j.1600-0501.2009.01848.x>
330 7.	Huynh-Ba G, Pjetursson BE, Sanz M, Cecchinato D, Ferrus J, Lindhe J, et al.
331 Analysis of the socket bone wall dimensions in the upper maxilla in relation to immediate
332 implant placement. Clin Oral Implants Res. 2010 Jan;21(1):37-42. Available from:

**Formatted:** Font: (Default) Times New Roman

333 <https://doi.org/10.1111/j.1600-0501.2009.01870.x>

**Formatted:** Font: (Default) Times New Roman

334 8.	Spray JR, Black CG, Morris HF, Ochi S. The influence of bone thickness on facial
335 marginal bone response: stage 1 placement through stage 2 uncovering. Ann Periodontol.
336 2000 Dec;5(1):119-28. Available from: <https://doi.org/10.1902/annals.2000.5.1.119>

**Formatted:** Font: (Default) Times New Roman

**Formatted:** Font: (Default) Times New Roman

337 9.	Kan JY, Rungcharassaeng K. Immediate placement and provisionalization of
338 maxillary anterior single implants: a surgical and prosthodontic rationale. Pract Periodontics
339 Aesthet Dent. 2000 Nov-Dec;12(9):817-24; quiz 26.
340 10.	Levine RA, Ganeles J, Gonzaga L, Kan JK, Randel H, Evans CD, et al. 10 Keys for
341 Successful Esthetic-Zone Single Immediate Implants. Compend Contin Educ Dent. 2017
342 Apr;38(4):248-260.
343 11.	Rosa AC, da Rosa JC, Dias Pereira LA, Francischone CE, Sotto-Maior BS.
344 Guidelines for Selecting the Implant Diameter During Immediate Implant Placement of a
345 Fresh Extraction Socket: A Case Series. Int J Periodontics Restorative Dent. 2016 May-
346 Jun;36(3):401-7. Available from: <https://doi.org/10.11607/prd.2381>

**Formatted:** Font: (Default) Times New Roman

**Formatted:** Font: (Default) Times New Roman

347 12.	Theisen FC, Shultz RE, Elledge DA. Displacement of a root form implant into the

**Formatted:** Font: (Default) Times New Roman

348 mandibular canal. Oral Surg Oral Med Oral Pathol. 1990 Jul;70(1):24-8. Available from:

**Formatted:** Font: (Default) Times New Roman

**Formatted:** Font: (Default) Times New Roman

349 <https://doi.org/10.1016/0030-4220(90)90172-o>

**Formatted:** Font: (Default) Times New Roman

350 13.	Kan JY, Roe P, Rungcharassaeng K, Patel RD, Waki T, Lozada JL, et al.
351 Classification of sagittal root position in relation to the anterior maxillary osseous housing for
352 immediate implant placement: a cone beam computed tomography study. Int J Oral
353 Maxillofac Implants. 2011 Jul-Aug;26(4):873-6.
354 14.	Ganz SD. Presurgical planning with CT-derived fabrication of surgical guides. J Oral
355 Maxillofac Surg. 2005 Sep;63(9 Suppl 2):59-71. Available from:

**Formatted:** Font: (Default) Times New Roman

**Formatted:** Font: (Default) Times New Roman

356 <https://doi.org/10.1016/j.joms.2005.05.156>

**Formatted:** Font: (Default) Times New Roman

357 15.	Fortin T, Champleboux G, Lormée J, Coudert JL. Precise dental implant placement in
358 bone using surgical guides in conjunction with medical imaging techniques. J Oral Implantol.
359 2000;26(4):300-3. Available from: https://doi.org/10.1563/1548-

**Formatted:** Font: (Default) Times New Roman

**Formatted:** Font: (Default) Times New Roman

360 1336(2000)026%3C0300:pdipib%3E2.3.co;2

**Formatted:** Font: (Default) Times New Roman

361 16.	Hebel KS, Gajjar RC. Cement-retained versus screw-retained implant restorations:
362 achieving optimal occlusion and esthetics in implant dentistry. J Prosthet Dent. 1997
363 Jan;77(1):28-35. Available from: <https://doi.org/10.1016/s0022-3913(97)70203-8>

**Formatted:** Font: (Default) Times New Roman

**Formatted:** Font: (Default) Times New Roman

364 17.	Du JK, Li HY, Wu JH, Lee HE, Wang CH. Emergence angles of the cementoenamel

**Formatted:** Font: (Default) Times New Roman

365 junction in natural maxillary anterior teeth. J Esthet Restor Dent. 2011 Dec;23(6):362-9.
366 Available from: <https://doi.org/10.1111/j.1708-8240.2011.00471.x>

**Formatted:** Font: (Default) Times New Roman

**Formatted:** Font: (Default) Times New Roman

367 18.	Linkevicius T, Vindasiute E, Puisys A, Peciuliene V. The influence of margin

**Formatted:** Font: (Default) Times New Roman

368 location on the amount of undetected cement excess after delivery of cement-retained implant
369 restorations. Clin Oral Implants Res. 2011 Dec;22(12):1379-84. Available from:

**Formatted:** Font: (Default) Times New Roman

**Formatted:** Font: (Default) Times New Roman

370 <https://doi.org/10.1111/j.1600-0501.2010.02119.x>

**Formatted:** Font: (Default) Times New Roman

371 19.	Michalakis KX, Hirayama H, Garefis PD. Cement-retained versus screw-retained
372 implant restorations: a critical review. Int J Oral Maxillofac Implants. 2003 Sep-
373 Oct;18(5):719-28.
374 20.	Garcia-Gazaui S, Razzoog M, Sierraalta M, Saglik B. Fabrication of a screw-retained
375 restoration avoiding the facial access hole: a clinical report. J Prosthet Dent. 2015
376 Nov;114(5):621-4. Available from: <https://doi.org/10.1016/j.prosdent.2015.06.007>

**Formatted:** Font: (Default) Times New Roman

**Formatted:** Font: (Default) Times New Roman

377 21.	Shreya Svitlana Anand, Ashok Velayudhan, Jaiganesh Ramamurthy. Immediate

**Formatted:** Font: (Default) Times New Roman

378 Implant Placement in Anterior Maxilla – A Retrospective Study. Int. J. Res. Pharm. Sci.
379 ~~[Internet]~~. 2020 Oct. 16 ~~[cited 2024 Jan. 6]~~;11((~~SPL~~ 3):1452-7. Available from:

**Formatted:** Font: (Default) Times New Roman

380 <https://ijrps.com/home/article/view/3098>

**Formatted:** Font: (Default) Times New Roman

**Formatted:** Font: (Default) Times New Roman

**Formatted:** Font: (Default) Times New Roman

**Formatted:** Font: (Default) Times New Roman

**Formatted:** Font: (Default) Times New Roman

**Formatted:** Font: (Default) Times New Roman

**Formatted:** Font: (Default) Times New Roman

**Formatted:** Font: (Default) Times New Roman

**Formatted:** Font: (Default) Times New Roman

**Formatted:** Font: (Default) Times New Roman

**Formatted:** Font: (Default) Times New Roman

**Formatted:** Font: (Default) Times New Roman

**Formatted:** Font: (Default) Times New Roman

**Formatted:** Font: (Default) Times New Roman

**Formatted:** Font: (Default) Times New Roman

381 22.	Cooper LF. Objective criteria: guiding and evaluating dental implant esthetics. J
382 Esthet Restor Dent. 2008;20(3):195-205. Available from: [https://doi.org/10.1111/j.1708-](https://doi.org/10.1111/j.1708-8240.2008.00178.x)
383 [8240.2008.00178.x](https://doi.org/10.1111/j.1708-8240.2008.00178.x)
384 23.	Januário AL, Duarte WR, Barriviera M, Mesti JC, Araújo MG, Lindhe J. Dimension
385 of the facial bone wall in the anterior maxilla: a cone-beam computed tomography study. Clin
386 Oral Implants Res. 2011 Oct;22(10):1168-1171. Available from:
387 <https://doi.org/10.1111/j.1600-0501.2010.02086.x>
388 24.	Buser D, Chappuis V, Belser UC, Chen S. Implant placement post extraction in
389 esthetic single tooth sites: when immediate, when early, when late? Periodontol 2000. 2017
390 Feb;73(1):84-102. Available from: <https://doi.org/10.1111/prd.12170>
391 25.	Chen K, Li Z, Liu X, Liu Q, Chen Z, Sun Y, Chen Z, Huang B ~~et al~~. Immediate
392 Implant Placement with Buccal Bone Augmentation in the Anterior Maxilla with Thin Buccal
393 Plate: A One-Year Follow-Up Case Series. J Prosthodont. 2021 Jul;30(6):473-480. Available
394 from: <https://doi.org/10.1111/jopr.13350>
395 26.	El-Askary AS. Use of connective tissue grafts to enhance the esthetic outcome of
396 implant treatment: a clinical report of 2 patients. J Prosthet Dent. 2002 Feb;87(2):129-32.
397 Available from: <https://doi.org/10.1067/mpr.2002.121405>
398 27.	Hürzeler MB, Zuhr O, Schupbach P, Rebele SF, Emmanouilidis N, Fickl S. The
399 socket-shield technique: a proof-of-principle report. J Clin Periodontol. 2010 Sep;37(9):855-
400 62. Available from: <https://doi.org/10.1111/j.1600-051x.2010.01595.x>
401 28.	Vindasiute E, Puisys A, Maslova N, Linkeviciene L, Peciuliene V, Linkevicius T.
402 Clinical Factors Influencing Removal of the Cement Excess in Implant-Supported
403 Restorations. Clin Implant Dent Relat Res. 2015 Aug;17(4):771-8. Available from:
404 <https://doi.org/10.1111/cid.12170>

**Formatted:** Font: (Default) Times New Roman

**Formatted:** Font: (Default) Times New Roman

**Formatted:** Font: (Default) Times New Roman

**Formatted:** Font: (Default) Times New Roman

**Formatted:** Font: (Default) Times New Roman

**Formatted:** Font: (Default) Times New Roman

**Formatted:** Font: (Default) Times New Roman

**Formatted:** Font: (Default) Times New Roman

**Formatted:** Font: (Default) Times New Roman

**Formatted:** Font: (Default) Times New Roman

405 29.	Pauletto N, Lahiffe BJ, Walton JN. Complications associated with excess cement
406 around crowns on osseointegrated implants: a clinical report. Int J Oral Maxillofac Implants.
407 1999 Nov-Dec;14(6):865-8.
408 30.	Testori T, Weinstein T, Scutellà F, Wang HL, Zucchelli G. Implant placement in the
409 esthetic area: criteria for positioning single and multiple implants. Periodontol 2000. 2018
410 Jun;77(1):176-196. Available from: <https://doi.org/10.1111/prd.12211>
411 31.	Buser D, Martin W, Belser UC. Optimizing esthetics for implant restorations in the
412 anterior maxilla: anatomic and surgical considerations. Int J Oral Maxillofac Implants.
413 2004;19 Suppl:43-61.
414 32.	Kim S-Y, Lim S-H, Gang S-N, Kim H-J. Crown and root lengths of incisors, canines,
415 and premolars measured by cone-beam computed tomography in patients with malocclusions.
416 Korean J Orthod. 2013 Dec;43(6):271-8. Available from:
417 <https://doi.org/10.4041/kjod.2013.43.6.271>

418

419 33.	Lee S-L, Kim H-J, Son M-K, Chung C-H. Anthropometric analysis of maxillary
420 anterior buccal bone Korean adults using cone-beam CT. J Adv Prosthodont. 2010
421 Sep;2(3):92-6. Available from: <https://doi.org/10.4047/jap.2010.2.3.92>
422 34.	Schiegnitz E, Al-Nawas B. Narrow-diameter implants: A systematic review and meta-
423 analysis. Clin Oral Implants Res. 2018 Oct;29 Suppl 16:21-40. Available from:
424 <https://doi.org/10.1111/clr.13272>
425 35.	Petrie CS, Williams JL. Comparative evaluation of implant designs: influence of
426 diameter, length, and taper on strains in the alveolar crest. A three-dimensional finite-element
427 analysis. Clin Oral Implants Res. 2005 Aug;16(4):486-94. Available from:
428 <https://doi.org/10.1111/j.1600-0501.2005.01132.x>
429 36.	Quek CE, Tan KB, Nicholls JI. Load fatigue performance of a single-tooth implant
430 abutment system: effect of diameter. Int J Oral Maxillofac Implants. 2006 Nov-

431

432

Dec;21(6):929-36.

37.	Erin K

**Formatted:** Border: Top: (No border), Bottom: (No border), Left: (No border), Right: (No border), Between : (No border)

433 Edmondson, Pedro M Trejo, Nikolaos Soldatos, Robin L Weltman. The ability to screw-
434 retain single implant-supported restorations in the anterior maxilla: A CBCT analysis. J
435 Prosthet Dent. 2022 Sep;128(3):443-449. Available from:

**Formatted:** Font: (Default) Times New Roman

436 <https://doi.org/10.1016/j.prosdent.2021.01.004>

**Formatted:** Font: (Default) Times New Roman

437 38.	Joseph Y K Kan, Kitichai Rungcharassaeng, Pongrapee Kamolroongwarakul, Guo-
438 Hao Lin, Hiroyuki Matsuda, Shi Yin, Hom-Lay Wang, Dennis Tarnow, Jaime L Lozada.
439 Frequency of screw-retained angulated screw channel single crown following immediate
440 implant placement and provisionalization in the esthetic zone: A cone beam computed
441 tomography study/ Clin Implant Dent Relat Res. 2023 Oct;25(5):789-794. Available from:

**Formatted:** Font: (Default) Times New Roman

442 <https://doi.org/10.1111/cid.13227>

**Formatted:** Font: (Default) Times New Roman

443

Response to Reviewers

To the Editor and reviewers of the PLOS ONE journal

Dear Editor and reviewers,

Thank you for taking the time to review my manuscript. I appreciate your feedback; they were accommodating. I have incorporated all of your suggestions in my revisitation as described in this letter.

## To Editor:

- All references have been reviewed and corrected to ensure completeness.
- Citation [37. Erin K Edmondson, Pedro M Trejo, Nikolaos Soldatos, Robin L Weltman. The ability to screw-retain single implant-supported restorations in the anterior maxilla: A CBCT analysis. J Prosthet Dent. 2022 Sep;128(3):443-449. Available from: <https://doi.org/10.1016/j.prosdent.2021.01.004>] has been added to line 273-274 in the discussion part of manuscript.
- Citation [38. Joseph Y K Kan, Kitichai Rungcharassaeng, Pongrapee Kamolroongwarakul, Guo-Hao Lin, Hiroyuki Matsuda, Shi Yin, Hom-Lay Wang, Dennis Tarnow, Jaime L Lozada. Frequency of screw-retained angulated screw channel single crown following immediate implant placement and provisionalization in the esthetic zone: A cone beam computed tomography study/ Clin Implant Dent Relat Res. 2023 Oct;25(5):789-794. Available from: <https://doi.org/10.1111/cid.13227>] has been added to line 274 and 278 in the discussion part of manuscript.

## Reviewer #2:

1. **Answer for using angulated screw channel of not: 548 teeth from 720 teeth (548/720) can be performed IIP in esthetic zone. The diameter of 3.3, 3.5, 4.1, 4.5 and 5 mm were used in all maxillary anterior teeth (central incisor, lateral incisor, canine). The diameter of 2.9 mm is used in lateral incisor only. The angulated screw channel was included in the calculation of all implant diameter except diameter of 2.9 mm was not included.**
   - The angulated screw channel was included in the calculation of all implant diameter except diameter of 2.9 mm was not included. The following sentence has been added to line 121-122

in the material and method part of manuscript. The remaining sentences are already included in line 118-121 in the material and method part of manuscript.

## 2. Answer for clarifying “limited restorability angulation for cement retained implant prosthesis": It is the most buccal angulation that allow for IIP in esthetic zone in cement retained implant prosthesis, which often in the middle of individual tooth, hence referred to as “middle half”. Even though, there are some possibilities to restore implant in more extreme buccal angulation with implant platform being more apical and palatal, there is an increased risk of complication

- The “middle half” has been explained for better comprehension and understanding in line 254-256 in the discussion part of manuscript.
3. **Answer for sample size calculation:**

**The purpose of this study is to determine the probability of IIP in esthetic zone using four different implant directions. This design of the study has not been performed yet. So, there will be no similarity in statistical data for this study.**

**The sample size calculation for estimating proportion will be used as shown in the formula (in Response to reviewers):**

**According to the retrospective study of Ashok Velayudhan et al. in 2020 that the objective related to the current study showed that in a total of 77 implants placed in the anterior maxilla, with 21 of them being immediate implants, 6 (or 7.8%) of 21 immediate implants were placed in the anterior maxilla alone.**

**The output of the sample size calculation is 114. The 120 patients are sufficient for our study. --> This must be mentioned in the manuscript.**

- This retrospective study of Ashok Velayudhan et al. has been mentioned and cited in line 92-93 in the material and method part of manuscript.

## 4. Answer for misaligned teeth as an exclusion criterion: Upper anterior teeth (canine- canine) misaligned is considered to exclusion criteria, which did not include in the sample size of our study.

- Misaligned upper anterior teeth has been added to exclusion criteria in line 104 in the material and method part of manuscript.
5. **Answer to elaborate on the methods used to determine the long axis of the tooth, the angulation/direction of the planned implant:**

**The line drawn from incisal edge to root apex was long axis of the tooth.**

**The point between incisal edge to CEJ was marked as point M. The point between the center of the most prominent point of cingulum to the incisal edge was marked as point S.**

- **In traditional cement retained implant prosthesis (incisal edge), long axis of implant will coincide with incisal edge.**
- **In traditional screw retained implant prosthesis (screw retained), long axis of implant will coincide with point S.**
- **In limit angulation for angulated screw channel (angle screw), long axis of implant will coincide at the point that an angle up to 25° from traditional screw retained.**
- **In limit restorability angulation for cement retained implant prosthesis (middle half), long axis of implant will coincide with point M.**
- All following sentences have been added to line 126-135 in the material and method part of manuscript.

## 6. Discussion

- The two mentioned studies have been added and compared to our study to line 273-289 in the discussion part of manuscript.

## 7. Answer related to the study limitation: In our study, to standardized the method, implant planning is limited to static predetermined angulation to anatomical relation and implant size. This could limited the probability as clinician can increase the depth of implant placement, modified implant angulation within restorable position. Future study is suggested to study the effect of probability associated with dynamic implant planning.

- All following sentences have been added to line 290-294 in the discussion part of manuscript.

Finally, I appreciate your feedback. They are accommodating and explicitly more reliable to my work. I am open to revising and improving my manuscript based on your suggestions. Thank you again for your time and consideration.

I look forward to your reply,

Sincerely,

Pranai Nakaparksin, DDS, MSD, FACP, FRCD(c)

Department of Advanced General Dentistry, Faculty of Dentistry, Mahidol University. 6 Yothi Street, Thung Phaya Thai, Rachathewi, Bangkok 10400 Thailand

Tel. +662 200 7853

Mobile. +66818088111

E-mail: [Pranai.nak@mahidol.ac.th](mailto:Pranai.nak@mahidol.ac.th)
